# TGF-Beta Modulates the Integrity of the Blood Brain Barrier In Vitro, and Is Associated with Metabolic Alterations in Pericytes

**DOI:** 10.3390/biomedicines11010214

**Published:** 2023-01-14

**Authors:** Leonie Schumacher, Rédouane Slimani, Laimdota Zizmare, Jakob Ehlers, Felix Kleine Borgmann, Julia C. Fitzgerald, Petra Fallier-Becker, Anja Beckmann, Alexander Grißmer, Carola Meier, Ali El-Ayoubi, Kavi Devraj, Michel Mittelbronn, Christoph Trautwein, Ulrike Naumann

**Affiliations:** 1 Molecular Neurooncology, Department of Vascular Neurology, Hertie Institute for Clinical Brain Research and Center of Neurology, University of Tübingen, 72076 Tübingen, Germany; 2 Department of Cancer Research (DOCR), Luxembourg Institute of Health (LIH), 1445 Strassen, Luxembourg; 3 Luxembourg Centre of Neuropathology (LCNP), 3555 Dudelange, Luxembourg; 4 Werner Siemens Imaging Center, Department of Preclinical Imaging and Radiopharmacy, University of Tübingen, 72076 Tübingen, Germany; 5 Mitochondrial Biology of Parkinson’s Disease, Department of Neurodegenerative Diseases, Hertie Institute for Clinical Brain Research and Center of Neurology, University of Tübingen, 72076 Tübingen, Germany; 6 Institute for Pathology and Neuropathology, University of Tübingen, 72076 Tübingen, Germany; 7 Department of Anatomy and Cell Biology, Saarland University, 66421 Homburg, Germany; 8 German Cancer Consortium (DKTK), 69120 Heidelberg, Germany; 9 Edinger Institute (Neurological Institute), Goethe University Hospital, 60528 Frankfurt am Main, Germany; 10 Luxembourg Centre for Systems Biomedicine (LCSB), University of Luxembourg, 4365 Esch-sur-Alzette, Luxembourg; 11 Department of Life Sciences and Medicine (DLSM), University of Luxembourg, 4365 Esch-sur-Alzette, Luxembourg; 12 Faculty of Science, Technology and Medicine (FSTM), University of Luxembourg, 4365 Esch-sur-Alzette, Luxembourg; 13 National Center of Pathology (NCP), Laboratoire Nationale de Santé (LNS), 3555 Dudelange, Luxembourg

**Keywords:** glioblastoma, blood–brain barrier, transforming growth factor beta, metabolomics

## Abstract

The blood–brain barrier (BBB) is a selectively permeable boundary that separates the circulating blood from the extracellular fluid of the brain and is an essential component for brain homeostasis. In glioblastoma (GBM), the BBB of peritumoral vessels is often disrupted. Pericytes, being important to maintaining BBB integrity, can be functionally modified by GBM cells which induce proliferation and cell motility via the TGF-β-mediated induction of central epithelial to mesenchymal transition (EMT) factors. We demonstrate that pericytes strengthen the integrity of the BBB in primary endothelial cell/pericyte co-cultures as an in vitro BBB model, using TEER measurement of the barrier integrity. In contrast, this effect was abrogated by TGF-β or conditioned medium from TGF-β secreting GBM cells, leading to the disruption of a so far intact and tight BBB. TGF-β notably changed the metabolic behavior of pericytes, by shutting down the TCA cycle, driving energy generation from oxidative phosphorylation towards glycolysis, and by modulating pathways that are necessary for the biosynthesis of molecules used for proliferation and cell division. Combined metabolomic and transcriptomic analyses further underscored that the observed functional and metabolic changes of TGF-β-treated pericytes are closely connected with their role as important supporting cells during angiogenic processes.

## 1. Introduction

The blood–brain barrier (BBB) is a highly selective barrier that prevents the non-selective transport of molecules from the blood into fluids of the central nervous system (CNS). It is formed by endothelial cells (EC) building the capillary walls, by pericytes that are embedded in the basal membrane, and is supported by astrocytes, the latter enrobing the capillary structure with their end feet. An intact BBB is highly important to maintain homeostasis in the central nervous system (CNS) since it allows the transport of molecules into the brain that are essential for its function. On the other hand, it protects the brain against pathogens. However, the BBB is also a major obstacle for the transport of drugs into brain tumors, at least in the infiltration zone of these tumors and for those tumor cells that deeply infiltrated the healthy brain and that are located far away from the original tumor (for review, see Ref. [1]). In neurological diseases, especially in glioblastoma (GBM), the most malignant brain tumor of adults, the BBB in and around the tumor core is leaky, leading to peritumoral edema and thus, to neurological deficits and a poor clinical outcome [2]. GBM-induced cerebral edema is currently treated with the corticosteroid dexamethasone (DEX) due to its ability to decrease BBB permeability. However, DEX, being an immunosuppressive drug, may be detrimental to immunotherapy in GBM patients [3]. In this regard, it is of central importance to understand why in GBM the BBB becomes disrupted and how this leakiness can be reduced or even prevented to avoid the use of corticosteroids.

Pericytes are mural cells adjacent to endothelial cells, wrapping around blood vessels. In the CNS, pericytes are necessary for the formation and regulation of the BBB. Under pathological conditions, such as in GBM, pericytes often undergo a functional change (reviewed in Ref. [4]). Previous metabolomics studies of pericytes have revealed their highly active energy metabolism, indicating the specific role of glucose and its uptake, ATP production from glucose catabolism, and fatty acid oxidation [5] which can be potentially dysregulated upon a pathology [6].

We have recently demonstrated that pericytes covering GBM-associated vessels (GA-Peris) can be distinguished from “normal” pericytes covering vessels outside the tumor area by their expression profile, especially by the expression of epithelial to mesenchymal transition (EMT) factors such as SLUG or TWIST [7]. We have further demonstrated that pericytes are prominently involved in the formation of GBM-associated vascular proliferations, and that GBM cells, by secretion of TGF-β, drive pericytes to change their growth morphology, induce proliferation and increase cell motility [8]. In consequence, we hypothesized that these functional alterations have an impact on BBB integrity. Using an in vitro BBB model containing primary brain pericytes and microvascular endothelial cells as well as glioma cells or astrocytes, we demonstrate that the treatment of tight endothelial cell/pericyte layers with TGF-β, or co-culture of these layers with TGF-β-secreting GBM cells, resulted in the disruption of this so far intact BBB.

Additionally, we employed ^1^H-NMR spectroscopy-based metabolomics and RNA-seq to determine the intracellular modulation of human brain microvascular pericytes (HBVP) to further underpin the biomolecular events taking place upon TGF-β treatment and identified significant alterations of their metabolism. Energy, growth, and several pathways were altered in HBVP upon TGF-β treatment compared to control, further indicating the fundamental changes in the pericyte’s environment.

## 2. Material and Methods

### 2.1. Cell Culture

T98G glioma cells, the latter expressing high levels of TGF-β [9] were from the American Type Culture Collection (ATCC, Manassas, VA, USA). SV-GA cells (a human astrocytic subclone of human fetal glial cells transduced with an origin-defective mutant of simian virus 40) were a kind gift of Walter Atwood (Brown University, Providence, RI, USA) and were described in Ref. [10]. SV-GA cells were tested for origin and integrity by staining against the astrocytic cells’ specific protein GFAP [11]. The cells were cultured, if not otherwise noticed, in DMEM containing 10% FCS, 1% penicillin, and 100 µg/mL streptomycin (P/S; both Sigma-Aldrich, Taufkirchen, Germany). Human brain vascular pericytes (HBVP) were purchased from ScienCell (Carlsbad, CA, USA, # 1201-prf) and were cultured, if not otherwise mentioned, on poly-L-lysine (PLL) coated plastic in pericyte medium (PM) in the presence of 2% FCS, 1% P/S and 1% pericyte growth supplements (PGS; containing apo-transferrin, insulin, EGF, FGF, insulin growth factor (IGF)-1 and hydrocortisone; all from ProVitro, Berlin, Germany). HBVP were used up to passage 8.

To determine the doubling time of T98G and SV-GA cells, 1000 cells were seeded in microtiter plates. After attachment, every 24 h hexadupletts of cells were stained with crystal violet as described [12]. Conditioned medium from T98G cells was prepared by seeding 200,000 cells. After attachment, the medium was changed to serum-free MCDB-131 medium (EC-Medium; Sigma-Aldrich) and collected 24 h later.

### 2.2. Isolation of Primary Porcine Brain Microvascular Endothelial Cells

All animal experiments in these studies were covered by the approval of the Regional Board Tübingen (notification to use animal organs for translational biomedical research, given to M. Schenk on 22 December 2017). Porcine brain microvascular endothelial cells (PBMVEC) were prepared from the domestic pig brains of animals housed in the Tübingen animal facility. The brains were collected and transported on ice in PBS containing 153 mM NaCl, 5.6 mM KCl, 1.7 mM CaCl_2_, 1.2 mM MgCl_2_, 15 mM HEPES, 0.01 g/mL BSA to the laboratory. The cells were isolated using a slightly modified protocol previously described [13]. The meninges were removed, and the cortex was cut into small pieces using scalpels, followed by homogenization. The tissue was digested in collagenase II (Life Technology) for 60 min at 37 °C, and purified by centrifugation in a 25% BSA solution, followed by a second enzyme treatment with collagenase/dispase (Roche) and DNase I for 50 min at 37 °C. The cells were then seeded in 10 cm dishes in MCDB-131 supplemented with 50 µg/mL endothelial cell growth supplements (ECGS; K. Devraj, Edinger Institute, Frankfurt/Main, Germany; [14]), 1% P/S, 2 mM L-glutamine, 0.01% heparin, and 0.01% sodium-bicarbonate (all from Sigma-Aldrich) if not otherwise stated. After 4–14 h, puromycin (Sigma-Aldrich) was added to the media for the next 2 days to obtain a pure culture of PBMVECs. Dead cells were removed by washing, and puromycin-free EC medium was added for two further days. Afterwards, the cells were collected and stored at −80 °C in FCS containing 10% DMSO. The purity and origin of endothelial cells were determined by staining the cells for the microvascular endothelial cell-specific protein ZO-1, the endothelial cell marker CD31, and claudin 5. Only the first passage of PBMVEC was used for experiments. During the experiments, the cells were cultivated in serum-depleted MCDB-131 supplemented with 1% PGS. All cells were cultured at 37 °C and 5% CO_2_ at 100% humidity.

### 2.3. The In Vitro BBB Model

To generate an intact and tight BBB in vitro, we used a modified protocol developed by Czupalla et al. [15]. PLL-coated Transwell inserts with a pore size of 0.33 µm were used (Thermo Fisher Scientific, Dreieich, Germany). 3300 HBVPs were seeded in a PM on the bottom side of the membrane and allowed to attach. Secondly, 35,000 PBMVEC were seeded on the top side of the membrane. The inserts were transferred into wells containing EC medium. At day 3 of culture, the medium was exchanged for freshly supplemented EC medium. The integrity of the barrier was assessed by transendothelial electric resistance (TEER) measurement using an EVOM-2 volt/ohm meter and an adjusted STX2 electrode chopstick (World Precision Instruments, Germany) as previously described [16]. After a strong barrier developed (generally after 5 days of culture), the medium in the lower chamber was exchanged for serum-deprived EC medium containing 1% of pericyte growth supplements (PGS), and recombinant human TGF-β1 and -β2 (PeproTech, Hamburg, Germany) were added at a final concentration of up to 12 ng/mL each. Alternatively, the inserts were transferred into new wells containing 200,000 T98G glioma or SV-GA astrocytic cells growing on the bottom. Before transferring the inserts, the medium of T98G or SV-GA cells was changed using serum-free EC medium. TEER measurements were performed every day using an EVOM2 volt/ohm meter with adapted chopsticks (World Precision Instruments, Friedberg, Germany). The impedance, and therefore the integrity, of the barrier, was calculated as described in Ref. [16] using the following equation: $TEER\;\left(Ω\times {\mathrm{cm}}^{2}\right)=R\;\left(Ω\right)\times A\;\left({\mathrm{cm}}^{2}\right)$. To block TGF-β activity, a pan-TGF-β neutralizing antibody (clone 1D11, # MA5-23795, ThermoFisher Scientific, Dreiech, Germany) was added to the TGF-β-containing medium in a 100-fold excess.

To determine the effect of TGF-β on BBB integrity in PBMVEC monocultures compared to PBMVEC/HBVP co-cultures, the cells were seeded as described above and 3 days later each 5 ng/mL TGF-β1 + -β2 was added. TEER measurement was then constantly measured over a period of 24 h on a CellZScope (NanoAnalytics, Münster, Germany) in accordance with the manufacturer´s protocol.

### 2.4. Immunocytochemistry and Immunofluorescence

For immunocytochemistry, PBMVECs were seeded on fibronectin-coated coverslips and allowed to attach. For staining for claudin 5 or ZO-1, the cells were fixed using 4% paraformaldehyde (PFA) and were permeabilized with 0.1% Triton X-100/PBS as previously described [11]. For staining with CD31, the cells were fixed with 10% formalin and permeabilized with ice-cold methanol according to the protocol supported by Novus (Novus Biologicals, Littleton, CO, USA). The following antibodies were used: anti-ZO-1 (#61-7300, rabbit polyclonal, Invitrogen/Thermo Fisher Scientific, 1:200); anti-claudin 5 (Invitrogen, Waltham, MA, USA, clone 4C3C2, mouse monoclonal, 1:100) and anti-CD31 (Biorad, Hercules, CA, USA, #MCA1746GA, mouse monoclonal, 1:100) For the detection of ZO-1, as secondary antibody a DL488-coupled anti-rabbit IgG for claudin 5 and CD31 expression, a DL488-conjugated goat anti-mouse-IgG (all from Thermo Fisher) was used. Immunofluorescence was performed on a Zeiss LSM 710 confocal microscope, using the ZEN20 software (Zeiss, Oberkochen, Germany).

### 2.5. Freeze Fracture Electron Microscopy

For electron microscopy, endothelial cells growing on the upper membrane side of in vitro BBB cultures were collected by mild trypsinization and scraping using a cell scraper. Cells from replicates were combined, then centrifuged, washed twice with PBS, and the cell pellet was subsequently prepared for freeze-fracture electron microscopy as described [17]. Alternatively, ECs were collected using a cell scraper, mounted between a sandwich of copper carriers, and plunge-frozen into a nitrogen-cooled liquid ethane/propane mixture using a Leica EM CPC (Leica Microsystems, Wetzlar, Germany). Afterwards, the sandwich carriers were transferred into a Leica EM BAF060 freeze-fracture and etching device. Samples were fractured at −162 °C and 1 × 10^−7^ mbar by chipping off the upper copper carrier, and subsequently deep etched for 5 min at −95 °C. Etched surfaces were shadowed with a 2 nm platinum-carbon coating applied at a 45° angle, stabilized by a layer of 20 nm carbon coating applied at 90°. The replicas were bound to a gold index grid using 0.5% Lexan polycarbonate plastic dissolved in dichloroethane (DCE). The DCE was allowed to evaporate at −20 °C overnight, attaching the replica to the grid. The samples were thawed at room temperature, and the carriers were removed. The replicas were cleaned by immersion in 70% sulfuric acid for 2 h, followed by 12% sodium hypochlorite (bleach) and double-distilled water for 1 h each. After drying, the Lexan film was removed by incubation in hot DCE. Analyses were performed using freeze-fracture replicas observed in a Zeiss EM 10 (Zeiss, Oberkochen, Germany) or an FEI Tecnai G2 transmission electron microscope (FEI, Thermo-Fisher Scientific, Munich, Germany) at 120 kV, equipped with a digital 8-bit camera. Tight junctions (TJ) were labeled manually. TJ density (total length of TJs) and complexity (number of TJ branches) were analyzed using ImageJ (Fiji, NIH, Bethesda, MD, USA [18]).

### 2.6. Metabolic Analysis

The mitochondrial to glycolytic ATP production was determined on a Seahorse™ XF96 Extracellular Flux Analyzer (Agilent Technology, Santa Clara, CA, USA) as previously described [19]. HBVP were seeded on poly-L-lysine coated Seahorse cell plates 24 h prior to the experiment and were treated as indicated. During the experiment, the oxygen consumption rate (*OCR*) and extracellular acidification rate (ECAR) were measured before any injection of mitochondrial toxins (refers to “basal state”). The wells were then injected sequentially with 8 µM oligomycin (Santa Cruz Biotechnology; refers to “minimal respiration”), 28 µM FCCP (Santa Cruz; refers to “maximal respiration”), and 40 µM antimycin A (Santa Cruz) plus 8 µM Rotenone (Sigma-Aldrich; refers to “mitochondrial inhibition”, and OCT/ECAR rates were measured again. Cell density was used for normalization. For this, after measurements, the cells were washed with PBS, permeabilized with Triton-X-100 (5% *w*/*v*, Sigma-Aldrich), and stained with 4,6-diamidin-2-phenylindol (DAPI, Sigma-Aldrich). Cell density was determined by fluorescence intensity on a Tristar LB942 fluorescence reader (Berthold Technologies, Bad Wildbad, Germany). Data analysis was performed using the Seahorse Software Wave, the XF Real-Time ATP Rate Assay Reporter Generator (both from Agilent Technologies, Santa Clara, CA, USA), followed by further analyses using Excel (v16.0 Microsoft Corporation, Redmond, WA, USA).

### 2.7. ^1^H-NMR Spectroscopy-Based Metabolomics of HBVP Intracellular Metabolites

After TGF-β treatment, the cells were washed twice with ice-cold PBS, and the adherent cells were shock-frozen with liquid nitrogen, placed on dry ice, and further on wet ice. After the addition of −80 °C cold methanol, the cells were scraped, collected, vigorously vortexed, and stored at −80° C until extraction. The metabolite extraction and measurement techniques have been previously described [19]. Metabolites were annotated and quantified by ChenomX NMR Suite 8.5 software (Chenomx, Edmonton, Alberta, Canada), which contains the additional human metabolome database (HMDB) 600 MHz library. All data were normalized by a reference sample with probabilistic quotient normalization (PQN) to account for dilution effects.

### 2.8. RNA Sequencing

For RNA-seq, HBVP were treated as indicated. Total RNA was prepared using the Nucleo Spin RNA Mini Isolation Kit with DNA Removal Column (Machery-Nagel, Düren, Germany). RNA-seq was performed at the c.ATG facility of the NGS Competence Center Tübingen (NCCT) using the QuantSeq 3′ mRNA-Seq Library Prep FWD and the NovaSeq 6000 SP Reagent Kit v1.5 with 200 cycles and 15 million clusters (both kits from Illumina, Berlin, Germany). Primary analyses were performed by sequencing data demultiplexing, and secondary analyses using the !Ensembl reference genome, followed by differential gene expression for the sample groups.

### 2.9. Combined Metabolomics and RNA-Seq Pathway Analysis

Metabolite and gene joint-pathway analysis was performed based on the Kyoto Encyclopedia of Genes and Genomes (KEGG) pathway database of integrated metabolic pathways that have both metabolites and metabolic genes. A combined query integration method using metabolite and gene fold change values from TGF-β-treated samples compared to control was performed via the MetaboAnalyst online platform [20]. A hypergeometric test was selected for enrichment analysis. Degree centrality was used as the topology measure.

### 2.10. Statistics

All BBB experiments were performed independently at least thrice with up to 10 technical replicates per experiment unless mentioned otherwise. Statistics on BBB experiments were done using GraphPad Prism 9.4.1. (Insight Partners, New York, NY, USA) or Excel v16.0 (Microsoft Corporation, Redmond, WA, USA). For TEER analyses, data from untreated cells served as control, and all replicates from each treatment group were combined for analyses. The *t*-test for unpaired samples, or the analysis of variance (ANOVA) test, followed by the Bonferroni post hoc test for multiple testing, was used. Results are represented as the mean ± standard error mean (SEM). *p*-values of <0.05 are considered statistically significant (ns: not significant; * *p* < 0.05, ** *p* < 0.01, *** *p* < 0.001, **** *p* < 0.0001). For metabolomics analysis, the MetaboAnalyst 5.0 web server (R-based online analysis tool, www.metaboanalyst.ca, accessed on 16 December 2022 [20]) was used with unpaired *t*-test analysis with a false discovery rate (FDR)-corrected *p*-value threshold of 0.05, and a fold change (FC) threshold of 1.2. Then, the student’s *t*-test was applied to compare metabolite concentrations between the control and TGF-β-treated cells using GraphPad Prism 9.3.1. software (Insight Partners, New York, NY, USA). We used principal component analysis (PCA), pattern hunter, heatmap, and joint-pathway analysis tools for data visualization. The heat map shows auto-scaled concentration values (−2;2) with clustered features generated by the Ward clustering algorithm based on Euclidean distance measure. Pattern hunter correlations are based on the Pearson r distance measure. Joint-pathway analysis is based on 84 metabolite sets based on KEGG human metabolic pathways (October 2019).

## 3. Results

### 3.1. Co-Cultures of Primary Endothelial Cells and Pericytes Develop a Tight BBB

**Figure 1:**
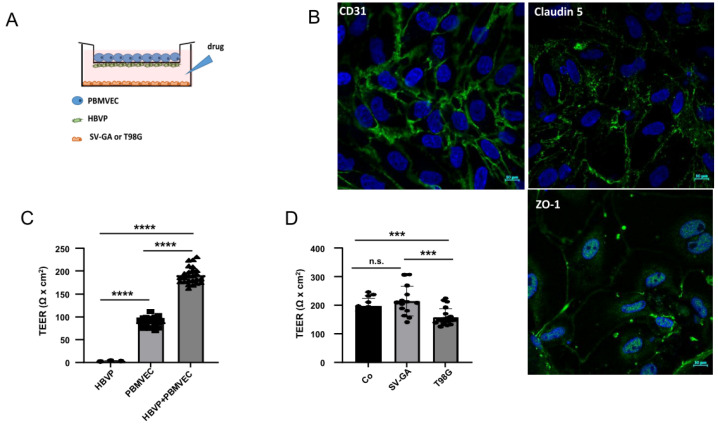
The in vitro blood–brain barrier (BBB) model. (**A**) Scheme of porcine brain microvascular endothelial cells (PBMVEC), human brain microvascular pericytes (HBVP) and SV40 large T-antigene immortalized astrocytic cells (SV-GA) or T98G glioblastoma cell line localization in the BBB co-culture model co-culture. (**B**) CD31, claudin 5 and ZO-1 staining of isolated PBMVEC (bars = 10 µm). (**C**) The cells were cultivated as described in the material and methods part either as monocultures (HBVP on the bottom insert membrane; PBMVEC on the top insert membrane) or as co-cultures. After 5 days, the membrane integrity was determined by TEER measurement (*n* = 3, each up to 10 replicates, SEM, *t*-test, **** *p* < 0.0001). (**D**) Co-culture of barrier-tight BBB-layers (containing PBMVEC and HBVP) with SV-GA or T98G cells. TEER was performed 24 h after co-culture (Co: no additional cells were seeded; *n* > 7, SEM, *t*-test, *** *p* < 0.005). Co-culture with astrocytic cells strengthens, with glioma cells weakens barrier integrity. (**C**,**D**) Circles, squares and triangles at the appropriate bars indicate data points of single experiments.

In our experiments, we used an in vitro BBB model that combines the culture of primary PBMVEC and HBVP and allows co-culture with other cells, the addition of conditioned medium, or the addition of cytokines such as TGF-β (Figure 1A). First, we validated the origin and purity of isolated PBMVEC by staining with CD31, claudin 5, and ZO-1. As shown in Figure 1B, PBMVECs provided a correct staining pattern for all three proteins. Isolated PBMVECs had a purity higher than 95%. PBMVECs alone developed a more leaky barrier, whereas after 5 days of co-culturing PBMVEC and HBVP, they created a tight barrier (Figure 1C, Supplementary Figure S1). To show that our model is feasible to measure exogenous effects influencing the barrier integrity, and as it is described that in vivo brain astrocytes positively, and glioma cells negatively influence the integrity of the BBB, we tested whether this can be reproduced in our model. For this, we transferred barrier-tight PBMVEC/HBVP co-culture membranes into cell culture wells containing immortalized astrocytic SV-GA or T98G GBM cells growing on the bottom of the well. These cell lines have been chosen to avoid artefacts induced by differences in cell proliferation of astrocytic and glioma cells since both SV-GA and T98G cells showed nearly equal doubling times (approximately 56 ± 4 h for SV-GA and 59 ± 6.6 h for T98G cells). As shown in Figure 1D, the co-culture of barrier-intact PBMVEC/HBVP layers with SV-GA cells further enhanced, whereas the co-culture with T98G cells significantly reduced the integrity of the barrier, indicating that the in vitro BBB model is feasible to determine influences on BBB integrity and leakiness.

### 3.2. TGF-β Treatment of HBVP Negativels Modelates the Integrity of the BBB

**Figure 2:**
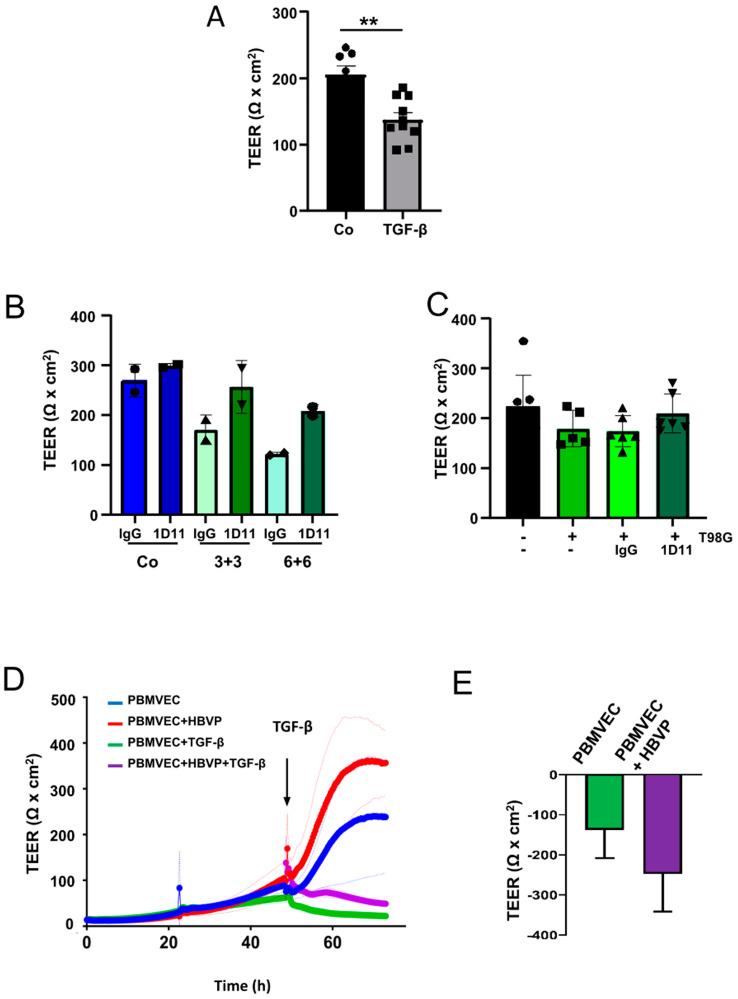
TGF-β negatively modulates the integrity of the BBB in vitro. (**A**) TGF-β negatively modulates the integrity of the barrier. Addition of TGF-β1 plus -β2 (each 5 ng/mL; grey bar) into the bottom chamber (HBVP cell membrane side) of barrier-tight BBB layers. Twenty-four hours later, the integrity of the barrier was determined by TEER (Co: sham treatment; black bar). *n* = 4, each 2 technical replicates, SEM, *t*-test, ** *p* < 0.01). (**B**) The cells were treated with TGF-β1 plus -β2 [each 3 ng/mL (3+3) or 6 ng/mL (6+6)] in the absence or presence of the pan-TGF-β neutralizing antibody 1D11 (1 µg/mL). Addition of 1D11 reverted the TGF-β mediated reduction in BBB integrity (Co: sham treatment, *n* = 2, each one replicate, SD). (**C**) Intact barrier-dense BBB layers were transferred into new wells containing either no cells (black bars) or T98G cells (green bars) as well as no antibody (-), 1D11 antibody, or an isotype control (IgG, each 1 µg/mL). Twenty-four hours later, TEER measurement was performed (*n* = 3, each 2 replicates, SEM, *t*-test). (**A**–**C**) Circles, triangles and squares at the appropriate bars present the data points of single experiments. (**D**) PBMVEC monoculture and PMBVEC/HBVP co-culture membranes were treated with TGF-β1 plus -β2 (each 5 ng/mL) 48 h after seeding. TEER measurement was performed as described in the Methods section (one out of three independent experiments is shown, light-colored lines indicate the SD of technical replicates). (**E**) Differences in the reduction in TEER in PBMVEC mono- and PBMVEC/HBVP co-cultures after addition of TGF-β1 plus -β2 (each 5 ng/mL; *n* = 3, SD, *p* = 0.18).

We have recently published that TGF-β modulates the function of HBVP. It induces cell motility and proliferation, and additionally modulates the pericytic growth pattern and morphology [8]. In this regard, we were interested in whether these changes are associated with the breakdown of the BBB. For this, we added TGF-β into the bottom chamber of wells containing barrier-intact PBMVEC/HBVP layers or transferred these cultures into new cell culture wells containing T98G cells growing on the bottom. T98G cells were chosen since these cells secrete high amounts of both TGF-β1 and -β2 [9]. The specificity of TGF-β as a modulator of BBB integrity was determined by the addition of the TGF-β neutralizing antibody 1D11. Addition of TGF-β1 plus -β2 at a concentration of >5 ng/mL each resulted in a significant decrease in the barrier density (Figure 2A,B). A trend towards a reduced barrier density was also observed if barrier-tight PBMVEC/HBVP membranes were transferred into wells containing T98G GBM cells (Figure 2C). The barrier leakiness induced by TGF-β or co-culturing the membranes with T98G GBM cells could be, at least partially, reverted by the addition of 1D11 (Figure 2B,C). We next examined whether the protective and supporting function of HBVP on barrier integrity was influenced by TGF-β. For this, we compared the effect of TGF-β on PBMVEC monocultures with that on PBMVEC/HBVP co-cultures but already added TGF-β 48 h after seeding, a time point a dense BBB was not completely built up. As shown in Figure 2D,E, the addition of TGF-β inhibited barrier tightening both in mono- and co-cultures. In addition, the supporting function of pericytes in the development of barrier tightness was reduced to some extent by TGF-β as shown by the additional TEER reduction in co-cultures (Figure 2E).

**Figure 3:**
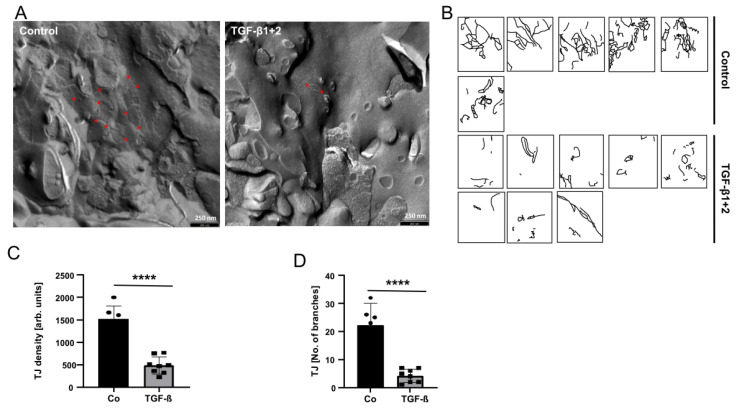
TGF-β treatment of HBVP reduced the amount and complexity of TJ in endothelial cells. (**A**) TGF-β1 plus -β2 (each 10 ng/mL) were added into the bottom chamber of wells containing intact BBB layers. Twenty-four hours later, PBMVEC growing on the upper part of the membrane were collected and prepared for freeze fraction electron microscopy. Red stars exemplarily indicate TJ branches. Exemplarily, one photograph is shown. (**B**) TJs were manually labeled in different visual fields (*n* = 2, in total 4 replicates). (**C**,**D**) Quantification of TJs density (**C**) and complexity (**D**). Black bar and circles present controls, grey bars and squares present the TGF-β treatment (*n* = 6 visual fields for controls, *n* = 8 for TGF-β; **** *p* < 0.0001).

To structurally demonstrate the effect of TGF-β on the BBB breakdown, we isolated PBMVECs growing on the upper HBVP/PBMVEC co-culture membrane 24 h after TGF-β treatment and determined the amount of tight junctions (TJ), typical structures of endothelial cells at an intact BBB, by freeze-fracture electron microscopy. The amount of TJ density and complexity in PBMVEC was notably reduced 24 h after the addition of TGF-β (Figure 3).

### 3.3. TGF-β Treatment Leads to a Distinct Metabolite Profile, Pathway Alterations, and Gene Expression Patterns in HBVP

**Figure 4:**
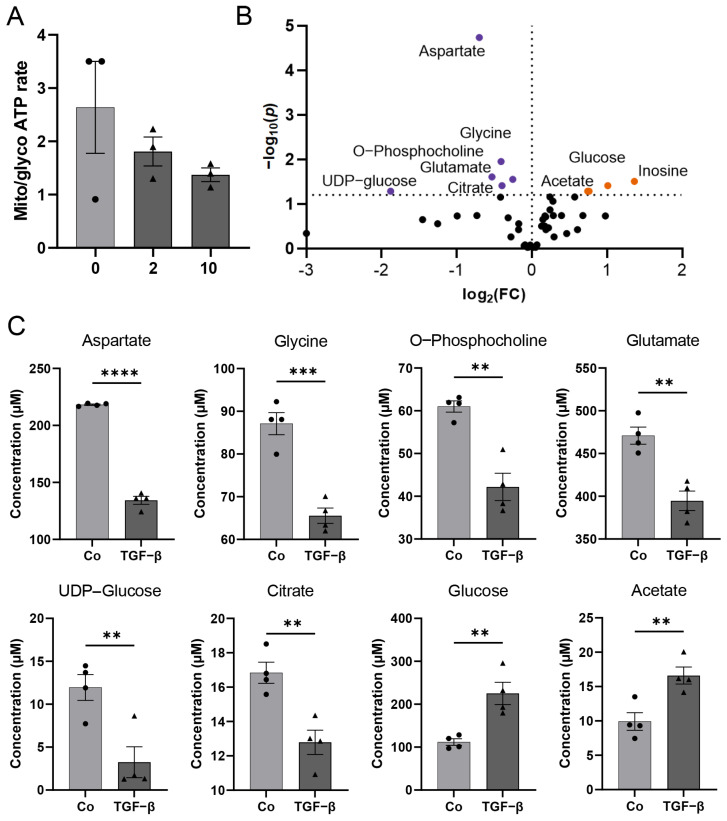
Metabolomics analysis of HBVP upon TGF-β treatment. (**A**) Determination of mitochondrial to glycolytic ATP production at different TGF-β concentrations using respiratory analysis. HBVP were treated with TGF-β (2 or 10 ng/mL, dark grey bars; black triangles) for 16 h or were left untreated (0; light grey bar; black dots; *n* = 3, each 6 technical replicates, SEM). (**B**) Volcano plot visualizing most significant metabolite concentration increase (orange dots) and decrease (purple dots) upon TGF-β treatment compared to control for *p* < 0.05, false discovery rate (FDR)-corrected data with fold change (FC) threshold >1.2 (*n* = 4). (**C**) Bar plots with individual replicate points and SEM of the most significant metabolite changes upon TGF-β treatment (each 5 ng/mL of TGF-β1 plus β2; black triangles) (*n* = 4): aspartate, glycine, O-phosphocholine, glutamate, UDP-glucose, and citrate concentrations were decreased compared to control (black dots), while glucose and acetate concentrations increased upon TGF-β treatment, based on parametric, unpaired *t*-test, ** *p* < 0.01, *** *p* < 0.001, **** *p* < 0.0001. Grey bar and circles present controls, dark grey bars and squares present the TGF-β treatment.

Recent studies have revealed the relevance of metabolic pathways in endothelial and mural cells, which control vascularization. The contribution of specific metabolic programs to the regulation of cell-state decisions has been extensively investigated in endothelial cells (for review, see Ref. [21]). However, metabolic changes in pericytes, especially in GBM-associated pericytes, have undergone very limited analysis to date. We primarily observed changes in the metabolic activity of pericytes that were treated with TGF-β, this was associated with an elevated proliferation and motility, with morphological alteration of these cells [8] as well as with the breakdown of an intact barrier (Figure 2). As it has been described that the activation of peripheral pericytes was associated with changes in energy generation, especially the switch of ATP production by oxidative phosphorylation towards glycolysis [22], we first determined mitochondrial and glycolytic ATP production upon TGF-β treatment. Indeed, a trend toward glycolytic ATP production was observed (Figure 4A). Further ^1^H-NMR spectroscopy-based metabolomics analysis quantified a total of 42 metabolites whose concentration was altered by TGF-β in HBVPs (Figure 4B,C, Supplementary Figure S2). From those, 9 metabolites, namely aspartate, glycine, O-phosphocholine, glutamate, UDP-glucose, citrate, glucose, acetate, and inosine concentration changes, were identified as statistically significant by univariate statistical analysis with *p* < 0.05 (FDR approved) and fold change threshold >1.2, when comparing TGF-β-treated cells to control (Figure 4B,C and Supplementary Figure S2D). Furthermore, 6 metabolites, namely pyroglutamate, tyrosine, lysine, alanine, leucine (the branched-chain amino acids), and NAD^+^, had altered concentrations based on an unpaired *t*-test (Supplementary Figure S1).

**Figure 5:**
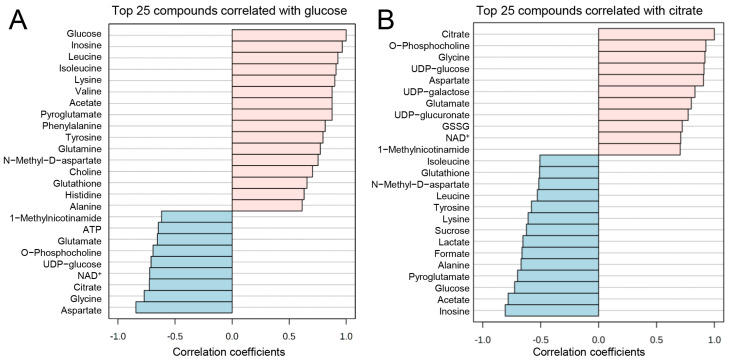
Pattern hunter analysis of glucose and citrate. (**A**) Glucose concentration pattern changes positively correlate (pink bars) with inosine, leucine, isoleucine, lysine, valine, acetate, pyroglutamate, phenylalanine, tyrosine, glutamine, N-methyl-D-aspartate, choline, glutathione, histidine, and alanine, while negatively correlate (blue bars) with aspartate, glycine, citrate, nicotinamide adenine dinucleotide (NAD)^+^, uridine diphosphate (UDP)-glucose, O-phosphocholine, glutamate, adenosine triphosphate (ATP) and 1-methylnicotinamide. (**B**) Citrate positively (pinks bars) correlates with O-phosphocholine, glycine, UDP-glucose, aspartate, UDP-galactose, UDP-glucuronate, oxidized glutathione (GSSG), NAD^+^, and 1-methylnicotinamide; and negatively (blue bars) correlates with inosine, acetate, glucose, pyroglutamate, alanine, formate, lactate, sucrose, lysine, tyrosine, leucine, N-methyl-D-aspartate, glutathione, and isoleucine concentration patterns, based on Pearson r distance measure (*n* = 4, two-group comparison).

We further investigated the correlation patterns (Spearman’s R) between the metabolites in our quantified dataset. Pattern hunter analysis of the glycolysis metabolite glucose revealed that inosine, leucine, isoleucine, valine (branched-chain amino acids), lysine, acetate, pyroglutamate, phenylalanine, tyrosine, and glutamine concentrations follow the same pattern in the dataset as glucose. Meanwhile, aspartate, glycine, citrate, NAD^+^, UDP-glucose, O-phosphocholine, glutamate, and ATP negatively correlated with glucose (Figure 5A). Furthermore, when correlated with the TCA cycle metabolite citrate, O-phosphocholine, glycine, UDP-glucose, aspartate, UDP-galactose, UDP-glucuronate, oxidized glutathione (GSSG), NAD+, and 1-methylnicotinamide were positively correlated to citrate concentration (Figure 5B). Inosine, acetate, glucose, pyroglutamate, alanine, formate, lactate, sucrose, lysine, tyrosine, leucine, N-methyl-D-aspartate, glutathione, and isoleucine negatively correlated to citrate concentration patterns in the dataset (Figure 5B).

In line with metabolomics analysis, RNA sequencing identified alterations in the expression pattern of genes that are involved in the regulation of energy generation and metabolic pathways, such as proline rich 5 (PRR5L, 4.9-fold upregulated by TGF-β), solute carrier family 46, member 3 (SLC46A3, 4.9 fold upregulated by TGF-β), peroxisome proliferator-activated receptor gamma, coactivator 1 alpha (PPARGC1AC, 3.2 × down), NADPH oxidase 4 (NOX4, 5.9 × up). Additionally, several genes important for the maintenance of the BBB such as the transcription factor 1 forkhead box S1 (FOXS1, 8.2 × up), the G protein-coupled receptor 183 (GPR183, 7.1 × up), and sparc/osteonectin, cwcv, and kazal-like domains proteoglycan 1/testican (SPOCK1, 2 × up), or that are indicators for disfunctional blood vessels or an impaired BBB (e.g., ADAM metallopeptidase with thrombospondin type 1 motif, 6, ADAMTS6, 2.6 × up; endothelial cell-specific molecule 1/endocan, ESM1, 7.6 × up) have been identified to be differentially regulated by TGF-β. In addition to metabolic genes and genes that are involved in BBB integrity, several genes are differentially expressed that are involved in processes of the EMT and EMT-associated processes such as cell migration, invasion, and proliferation (Supplementary Tables S1 and S2).

### 3.4. Joint-Pathway Analysis Illustrates Further Pathways Dysregulated upon TGF-β Treatment

**Figure 6:**
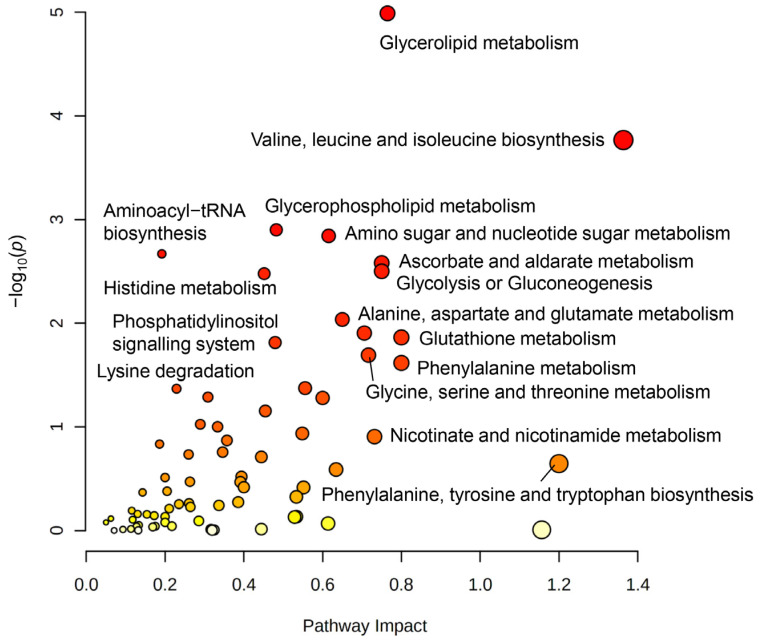
Joint pathway analysis of ^1^H-NMR spectroscopy-based metabolomics and RNA-seq gene fold change data. Pathway analysis indicates the most significantly changed metabolic pathways (the node color scheme is based on *p*-values from yellow dots = low significance to red dots = high significance and the node radius is determined based on the pathway impact value). Glycerolipid metabolism, branched-chain amino acid (BCAA) metabolism of valine, leucine, and isoleucine biosynthesis, glycerophospholipid metabolism, amino sugar and nucleotide sugar metabolism, ascorbate metabolism, glycolysis or gluconeogenesis, and alanine, aspartate, and glutamate metabolism, were most affected based on Kyoto Encyclopedia of Genes and Genomes (KEGG) pathway database. For joint-pathway analysis, data from metabolomics (*n* = 4) and RNA-seq (*n* = 4) were used.

Finally, we performed a joint-pathway analysis combining metabolite and gene expression data, using fold change values of 42 metabolites and genes that were obtained from TGF-β treatment samples and compared to the control. By using the MetaboAnalyst 5.0 online tool (Wishard Research Group, University of Alberta, Canada) for joint-pathway analysis [20], and selecting human organism (homo sapiens), we identified 9 significantly changed pathways: glycerolipid metabolism, valine, leucine, and isoleucine biosynthesis (branched-chain amino acid (BCAA) biosynthesis), glycerol-phospholipid metabolism, amino sugar and nucleotide sugar metabolism, aminoacyl-tRNA biosynthesis, ascorbate and aldarate metabolism, glycolysis or gluconeogenesis, histidine metabolism, and alanine, aspartate, and glutamate metabolism (Figure 6, Supplementary Table S3). Joint-pathway analysis of differentially regulated genes and metabolites indicated a close network of pathways involved in processes of cell growth that could be connected to angiogenesis, vessel structure, EMT, and proliferation, as well as energy and redox metabolism.

## 4. Discussion

The regulation of BBB integrity by pericytes in GBM is currently of great interest, as pericytes are functionally modulated and activated by glioma cells [8,23,24]. Well-established protocols for the isolation of brain microvascular pericytes and endothelial cells open the possibility of studying the pericyte influence on BBB integrity in vitro [25,26]. TEER measurement revealed a barrier integrity-increasing effect, finally resulting in a tight barrier, when PBMVEC, described to be feasible cells to study BBB integrity in vitro [27], separated by a 0.33 µm pore membrane, were co-cultured with HBVP. Only a small barrier-increasing effect was achieved if astrocytes were presented, yet a significant decrease, if TGF-β secreting GBM cells were presented to the membranes during the development of the barrier (Figure 1D). One limitation of our model might be the cross-species usage of porcine endothelial cells and human pericytes. In this respect, none of the momentarily available human in vitro BBB models meet all the criteria for in vivo BBB models. Furthermore, cross-species BBB models have been successfully used in the past [28]. We believe that our data show the feasibility of the in vitro BBB model to determine whether GBM-secreted factors will influence BBB integrity.

We have previously shown that glioma vessel-associated pericytes (GA-Peris) expressed key EMT factors such as SLUG and TWIST [7]. Additionally, TGF-β, a classical inductor of EMT processes, which is highly secreted by glioma cells, induced SLUG expression in HBVPs, in parallel with the induction of proliferation, cell motility, and alpha-smooth muscle cell actin (αSMA) expression, resulting in the activation of pericytes [7,8,29]. This opened the discussion of whether these TGF-β-mediated functional changes and activation of pericytes influence BBB integrity. Thanabalasundaram et al. have shown that pericytes differentiated in the presence of TGF-β and subsequently co-cultured with endothelial cells were not able to establish a tight BBB [30]. In our present study, we demonstrate that not only the establishment of an intact BBB was disturbed by TGF-β but also the integrity of a so far intact barrier was destroyed, putatively by a TGF-β-mediated prohibition of the supporting function of pericytes in the maintenance of the BBB. Similar results were observed when T98G GBM cells were co-cultured adjacent to the pericytes. Our observations are in line with the observation of Atis et al., who recently published that inhibition of the TGF-β receptor type I prevented BBB disruption in hypertensive mice [31]. We observed reduced BBB integrity when we cultured “BBB-intact” inserts in the presence of T98G. The effects were less notable when adding recombinant TGF-β into the pericytic compartment. However, this might be explained by the fact that we exchanged the medium in the compartment containing T98G cells for serum-deprived EC medium just before transferring the BBB-dense inserts (Figure 1A). Therefore, no TGF-β was present at the beginning of the treatment. Whilst the TGF-β concentration produced by T98G cells increased during co-culture, it might be even low at the time point of measuring the barrier integrity 24 h later and might be also lower than the concentration of recombinant TGF-β1 plus -β2 we used when initially adding this cytokine to the lower compartment. The observed effects on BBB integrity were TGF-β-dependent since neutralization of TGF-β activity at least partially abolished this effect (Figure 2B,C). Since we only performed short-term TGF-β treatment of pericytes which is not sufficient to alter the differentiation state of these cells [30], our data suggest that there is a direct effect of TGF-β in endorsing pericyte barrier functions of endothelial cells in addition to its role in differentiation. This is strengthened by our observation that a TGF-β-mediated complete breakdown of the barrier was observed in both PBMVEC mono- as well as in PBMVEC/HBVP co-cultures, indicating that the supporting function of pericytes was also inhibited by TGF-β (Figure 2D,E). This is further supported by the observation that the amount and complexity of TJs were significantly reduced in co-cultured endothelial cells (Figure 3). However, we cannot completely exclude the direct effects of TGF-β on endothelial cells using barrier-tight PBMVEC/HBVP membranes for our analyses. Even if adding TGF-β to the pericytic compartment or co-culturing barrier-tight HBVP/PBMVEC membranes with glioma cells at the time point we measured an intact and tight barrier function (Figure 1C,D, Figure 2A–C and Supplementary Figure S1), there might be some additional TGF-β effects on PBMVECs.

It is well known that TGF-β is able to increase glycolysis in GBM cells (for a review, refer to Ref. [32]). Here, we identified significant changes in the concentrations of a variety of metabolites as well as in the expression of metabolic pathways associated with this phenomenon in TGF-β-treated HBVP. Based on the trend from mitochondrially towards glycolytically generated ATP in HBVP upon TGF-β treatment (Figure 4A,C, Supplementary Figure S2), we interpreted the changes of metabolites that were found to be significant with an FDR-adjusted *p*-value < 0.05. We hereby want to highlight the intracellular increase in glucose and decrease in citrate under TGF-β treatment as key findings as this supports a switch in energy production from mitochondrial oxidative phosphorylation (OxPhos) towards glycolysis. A similar observation has been previously described for activated peripheral non-brain pericytes, associated with barrier integrity ([22] and reviewed in Ref. [5]). The postulated TGF-β-mediated switch from OxPhos to glycolysis we observed in HBVP was further associated with altered concentrations of key metabolites from the pentose phosphate pathway (PPP), which is in line with the observed metabolic shift. The shift towards PPP subsequently feeds nucleotide synthesis necessary for proliferation and NADPH to maintain redox and counteract oxidative stress (Figure 4 and Figure 5 and Supplementary Figure S2). Furthermore, changes in glycerolipid and glycerophospholipid metabolism (also known as Kennedy pathway-related metabolites) and gene expression suggest enhanced cell membrane synthesis as well as amino-tRNA and BCAA biosynthesis pathway changes that point towards altered protein synthesis (Figure 5 and Figure 6). This fits well with our former observations that pericytes are the most prominent cells in GBM associated vascular proliferates, and that HBVP that underwent a TGF-β mediated EMT reprogramming showed elevated proliferation [7,8]. Alterations in phospholipid metabolism and the Kennedy pathway have previously been reported as markers for recurrent glioblastoma [33]. In addition, enzymes modulating the energy metabolism of cells are differentially regulated: SLC46A3 (4.9 × upregulated by TGF-β) has been suggested to be involved in the plasma membrane electron transport (PMET), a plasma membrane analog to the mitochondrial electron transport chain that contributes to energy production by supporting glycolytic ATP production [34]. PRR5L modulates mTORC2 activity, which is known to regulate glucose uptake, glycolysis, and the PPP [35]. Nicotinamide adenine dinucleotide phosphate oxidase (NOX)-4, 5.9 × upregulated by TGF-β, is highly upregulated in stroke-associated pericytes, and its overexpression in pericytes induces a breakdown of the BBB by upregulating matrix metalloproteinase (MMP)-9 [36], the latter also associated with capillary damage during ischemia [37]. In contrast, PPARGC1 mRNA, coding for PGC-1α, was 3.2-fold downregulated by TGF-β. PGC-1α is a transcriptional key regulator of energy metabolism and a master regulator for mitochondrial biogenesis [38]. This supports our observation that ATP production was shifted towards glycolysis by TGF-β in HBVP. Besides, PGC-1α participates in the regulation of carbohydrate and lipid metabolism, which in TGF-β-treated HBVP we also found to be modulated [39]. However, not only the energy metabolism of HBVP was modulated by TGF-β. In fact, increased concentrations of BCAA have been seen as important drivers of cell migration, and mTORC1 activation [40] with mTORC1 hyperactivation has been linked to the promotion of angiogenesis in endothelial cells [41], and an elevated migration of HBVP was observed in GA-Peris that underwent an EMT-like transition [8]. Pericytes are particularly sensitive to oxidative stress [42,43,44]. It would therefore be interesting to investigate the impact of a shift from OxPhos to glycolysis on the oxidative state in these cells. A first hint that metabolic changes in TGF-β-treated HBVP are connected to oxidative stress is highlighted by changes to the glutathione and GSSG levels (Supplementary Figure S2A).

Joint metabolomics and RNA-seq pathway analysis identified a network of pathways that are important for angiogenesis, vessel structure, cell motility, anabolic and catabolic processes, as well as EMT signaling, that were modulated in GA-Peris [7,8]. To better understand these processes in detail, further studies disturbing or blocking different metabolic, motility, or proliferation-associated pathways are necessary to unravel the detailed connections of changes in metabolite concentrations to (i) BBB integrity, (ii) vessel structure, (iii) pericyte activation, or (iv) motility, and to determine which of the alterations in GA-Peris is connected to modulations of (v) the vessel structure or (vi) to the BBB maintenance, or (vii) if it is the GBM-induced homeostasis breakdown in GA-Peris that is responsible for the tumor-associated chaotic vessel structure and BBB breakdown. This might be done by the intervention of different metabolic, motility, or proliferation-associated pathways. Additionally, the portion of TGF-β-induced changes in pericytes in regard to their supporting function in BBB maintenance has to be evaluated as it is known that TGF-β also has direct effects on endothelial cells [45]. This might be carried out by the deletion of TGF-β receptors in endothelial cells or by the disruption of the intracellular TGF-β signaling in these cells. Nevertheless, we believe that TGF-β further modulates BBB-supporting functions of pericytes as the effect of TGF-β on pericytes is similar to that on endothelial cells. In both cell types, TGF-β induces an “epi/endothelial” to mesenchymal transition signature [7,8,46]. However, another study points toward a protective role of TGF-β on brain endothelial cells, whereas a third report stated the opposite effects [47,48]. In the latter study, immortalized brain capillary endothelial cells were used. However, in our hands, these immortalized cells, in contrast to first-passage primary PBMVECs, failed to generate a tight, intact barrier when co-cultured with HBVP and therefore might not be an optimal model to measure even small effects on how BBB integrity can be influenced by GBMs.

This study provides novel insights into how metabolic changes in brain pericytes are associated with their function and might thus be used to develop new strategies targeting angiogenic processes to treat GBM. In this regard, one might think to normalize tumor vascularization, as this has been shown to improve glioblastoma outcomes [49] and might lead to a functional vessel structure, function, and an intact BBB. This, in turn, diminishes brain edema, an important neurological issue of GBM that makes the usage of corticosteroids indispensable, but on the other hand, interferes with recently developed immunotherapeutic approaches. Furthermore, an intact tumor vessel structure upon vessel normalization might improve the transport of BBB passable drugs into the tumor tissue [50]. Future in vitro and in vivo studies, including the perturbation of the metabolic pathways we identified in this study, are needed to unravel the complex mechanism of violated GBM angiogenesis and to develop strategies for vessel normalization.

## 5. Conclusions

In summary, our data indicate that glioma-secreted TGF-β not only results in dysfunctional and leaky new-built vessels during tumor-associated neoangiogenic processes but also leads to the disturbance of an intact BBB by modulating the supportive function of pericytes. Metabolic alterations in the pericytes are associated with the breakdown of the BBB, shifting the cells’ energy generation from OxPhos towards glycolysis, and leading to modifications in several metabolic-associated pathways. This putatively influences several functional processes in these cells that finally modulate their functional behavior. Further animal models will show whether the inhibition of TGF-β has an impact on BBB integrity in the tumor area also in vivo.

## Data Availability

The datasets generated during and/or analyzed during the current study are available on the Mendeley data repository (https://doi.org/10.17632/17632/8r4wph5g7z.1).

## Supplementary Materials

The following supporting information can be downloaded at: https://www.mdpi.com/article/10.3390/biomedicines11010214/s1, Supplemental data for this article are available as 2 supplementary figures and 3 supplementary tables. Figure S1: PBMVEC and HBCPs were seeded on membranes as described in the Materials and Methods section. The tightness of the barrier that was generated was determined by measuring TEER. A stable and dense barrier was generated 5 days after coculture (** *p* < 0.01; *n* = 3; two to six replicates each, SD). Figure S2: Metabolomics analysis of HBVP upon TGF-β treatment. (A) Heatmap of all quantified metabolites in VBHP, illustrated as auto-scaled concentration values (−2;2), features clustered by Ward clustering algorithm based on Euclidean distance measure. (B) Principal component analysis (PCA) scores plot illustrating the treatment and sample group separation based on different metabolic profile, PC 1 61.8%, PC 2 25.4%. (C) PCA bi-plot indicates specific metabolites that drive the group separation: aspartate and glutamate are more associated with control group, while glutamine, pyroglutamate, glucose, lactate, and formate are more associated with the TGF-β treatment group. (D) Bar plots visualizing individual replicate points and SEM of significant metabolite changes upon TGF-β treatment (black triangles), in addition to Figure 4C: inosine, pyroglutamate, tyrosine, lysine, alanine, and leucine concentrations were increased compared to control (black dots), while NAD^+^ concentration was reduced in TGF-β treatment group based on parametric, unpaired *t*-test, * *p* < 0.05, ** *p* < 0.01. (E) Volcano plot visualizing most significant metabolite increased concentrations (orange dots) and decreased concentrations (purple dots) upon TGF-β treatment compared to control for *p* < 0.05, with fold change (FC) threshold 0.8. Table S1: Upregulated genes with a log fold change of > 2 in TGF-β-treated HBVP. Questions marks indicate functions that are only suggested to be associated with the appropriate gene. Table S2: Downregulated genes with a log fold change of >2 in TGF-β-treated HBVP. Questions marks indicate functions that are only suggested to be associated with the appropriate gene. Table S3: Joint-pathway analysis of metabolomics and RNA sequencing datasets. Most significantly changed pathways of TGF-β vs. control, based on joint-pathway analysis by metabolite concentrations and gene expression fold change, top 14 metabolic pathways.

## Abbreviations

ADAMTS6	ADAM metallopeptidase with thrombospondin type 1 motif, 6
AJ	adherent junction
ANOVA	analysis of variance
BBB	blood–brain barrier
BCAA	branched amino acid
BMVEC	brain microvascular endothelial cells
BSA	bovine serum albumin
CM	conditioned medium
CNS	central nerve system
DMEM	Dulbecco’s Modified Eagle’s Medium
ECGS	endothelial cell growth supplements
EGF	epidermal growth factor
EM	epithelial cell growth medium
EMT	epithelial-to-mesenchymal transition
ESM1	endothelial cell-specific molecule 1/endocan
FC	fold change
FCS	fetal calf serum
FGF	fibroblast growth factor
FDR	false discovery rate
FOXS1	forkhead box S1
GBM	glioblastoma
GJ	gap junction
GPR183	G protein-coupled receptor 183
HBVP	human brain microvascular pericytes
HRP	horseradish peroxidase
IGF-1	insulin-like growth factor 1
KEGG	Kyoto Encyclopedia of Genes and Genomes
mTORC1	mammalian target of rapamycin complex 1
NOX4	NADPH oxidase 4
NVU	neurovascular unit
o/n	overnight
OxPhos	oxidative phosphorylation
PBMVEC	porcine brain microvascular endothelial cells
PCA	principal component analysis
PGS	pericyte growth supplement
PLL	poly-L-lysine
PM	pericyte medium
PMET	plasma membrane electron transport
PPARGC1AC	peroxisome proliferator-activated receptor gamma, coactivator 1 alpha
PRR5	proline rich 5
P/S	penicillin/streptomycin
RT	room temperature
SLC46A3	solute carrier family 46, member 3
SD	standard deviation
SEM	standard error of the mean
SPOCK1	sparc/osteonectin, cwcv and kazal-like domains proteoglycan 1
SV-GA	SV40 large T-antigene immortalized astrocytic cells
TEER	transendothelial electric resistance
TGF-β	transforming growth factor beta
TJ	tight junction
VEGF	vascular endothelial growth factor
ZO	zonula occludens
