# Supplementary material for: TGF-Beta Modulates the Integrity of the Blood Brain Barrier In Vitro, and Is Associated with Metabolic Alterations in Pericytes
Source: Biomedicines. 2023 Jan 14;11(1):214. doi: 10.3390/biomedicines11010214 (PMC9855966; doi:10.3390/biomedicines11010214)
Supplement: Supplementary file 1 [file biomedicines-11-00214-s001.zip › Supplementary Figure 1.pdf]

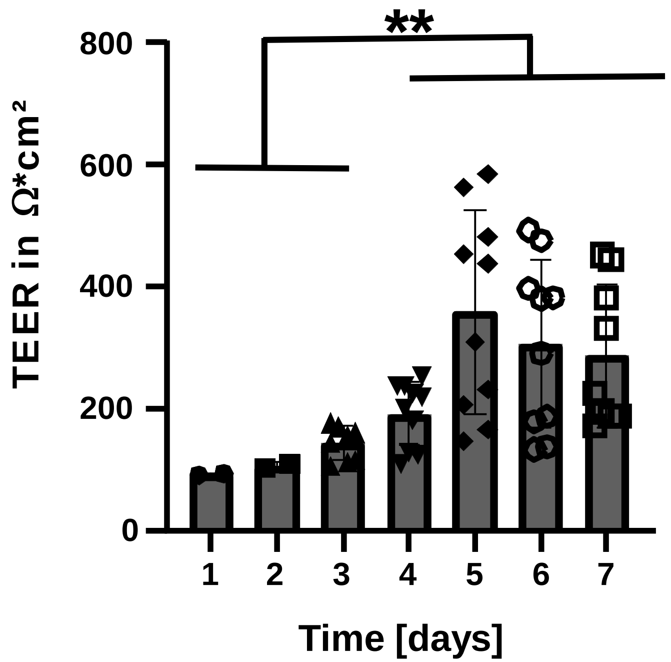

Supplementary Figure S1: PBMVEC and HBCPs were seeded on membranes as described in the Material and Methods part. The tightness of the barrier that was generated was determined by measuring TEER. A stable and dense barrier was generated 5 days after coculture (n=3, two to six replicates each, SD).
