# Supplementary material for: TGF-Beta Modulates the Integrity of the Blood Brain Barrier In Vitro, and Is Associated with Metabolic Alterations in Pericytes
Source: Biomedicines. 2023 Jan 14;11(1):214. doi: 10.3390/biomedicines11010214 (PMC9855966; doi:10.3390/biomedicines11010214)
Supplement: Supplementary file 1 [file biomedicines-11-00214-s001.zip › Supplementary Figure 2.pdf]

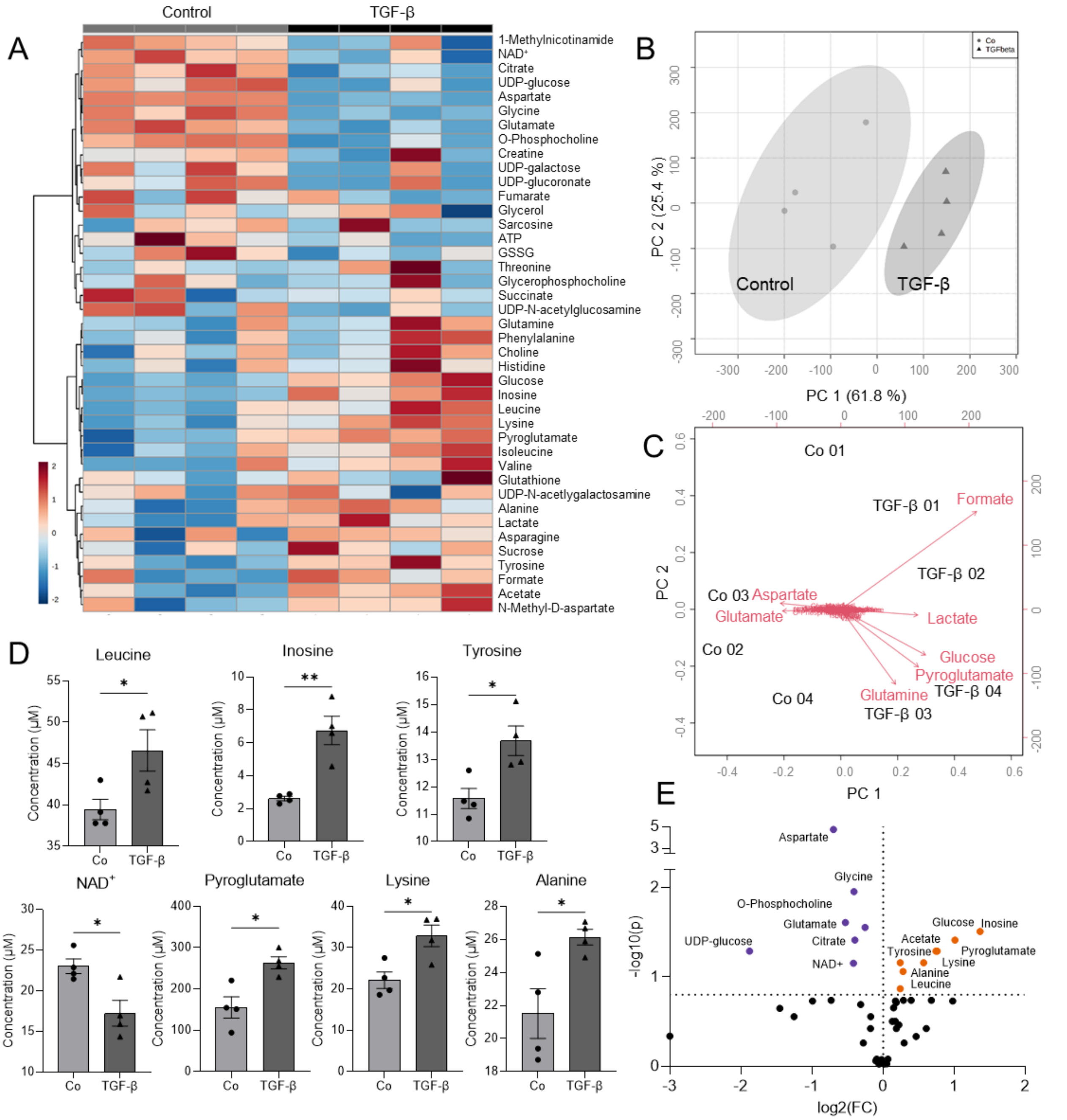

Supplementary Figure S2: Metabolomics analysis of HBVP upon TGF-β treatment. (**A**) Heatmap of all quantified metabolites in VBHP, illustrated as auto scaled concentration values (-2;2), features clustered by Ward clustering algorithm based on Euclidean distance measure. (**B**) Principal component analysis (PCA) scores plot illustrating the treatment and sample group separation based on different metabolic profile, PC 1 61.8%, PC 2 25.4%. (**C**) PCA bi-plot indicates specific metabolites that drive the group separation: aspartate and glutamate are more associated to control group, while glutamine, pyroglutamate, glucose, lactate and formate are more associated with the TGF-β treatment group. (**D**) Bar plots with visualizing individual replicate points and SEM of significant metabolite changes upon TGF-β treatment (black triangles), in addition to Figure 4 C: inosine, pyroglutamate, tyrosine, lysine, alanine and leucine concentrations were increased compared to control (black dots), while NAD^+ concentration was reduced in TGF-β treatment group based on parametric, unpaired t-test, * $p < 0.05$, ** $p < 0.01$. (**E**) Volcano plot visualizing most significant metabolite increased concentrations (orange dots) and decreased concentrations (purple dots) upon TGF-β treatment compared to control for $p<0.05$, with fold change (FC) threshold 0.8.
