# Supplementary material for: TGF-Beta Modulates the Integrity of the Blood Brain Barrier In Vitro, and Is Associated with Metabolic Alterations in Pericytes
Source: Biomedicines. 2023 Jan 14;11(1):214. doi: 10.3390/biomedicines11010214 (PMC9855966; doi:10.3390/biomedicines11010214)
Supplement: Supplementary file 1 [file biomedicines-11-00214-s001.zip › Supplementary Table 1.pdf]

Supplementary Table S1: Upregulated genes with a log fold change of > 2 in TGF-β treated HBVP

Symbol	Gene Name	Log fold change	Functions	Associated with	Reference
FOXS1	forkhead box S1	8.25 ↑	• Promotes cell proliferation and EMT in gastric cancer.<br>• TGFβ-induced FOXS1 controls EMT.<br>• Forkhead proteins required for brain pericyte differentiation, development and maintenance of the BBB.	TGF-β<br>proliferation<br>cell motility<br>EMT<br>Cancer<br>pericyte development<br>BBB	Sci Rep. 2019 (1):5281.<br>doi: 10.1038/s41598-019-41717-w<br>Hepatology Comm. 2022, 6 (5): 1157<br>Dev Cell 2015, 6;34(1): 19
ESM1	endothelial cell-specific molecule 1 /Endocan	7.60 ↑	• Specific biomarker of tip cells during neo-angiogenesis.<br>• Expression increased by pro-angiogenic growth factors.<br>• Role in endothelium-dependent pathological dis-orders.<br>• Secreted proteoglycan that has been shown to indicate angiogenic activity.<br>• Putative indicator of new blood vessels that are not functional.	angiogenesis<br>cancer<br>BBB (?)	Front Oncol. 2021;11: 687120.<br>Immunol Methods 2013;398-399:27
COMP5/TSP5	cartilage oligomeric matrix protein/thrombospondin 5	7.20 ↑	• Extracellular matrix protein.<br>• Member of the TSP family.<br>• Cell adhesion.<br>• Induced by TGF-ß<br>• Remodeling of the tumor microenvironment	cell motility<br>cancer<br>angiogenesis<br>TGF-β	JBC 2005, 280;(38): 32655.<br>Arteriosclerosis, Thrombosis, and Vascular Biology. 2001;21:47<br>Vessel Plus. 2018;2:30.
GPR183	G protein-coupled receptor 183	7.13 ↑	• Regulates migration of astrocytes.<br>• Communication between astrocytes and macrophages.<br>• Suggested to be involved in the regulation of the neuro-vascular unit.<br>• G protein coubeld receptors are essential for the integity of the BBB.	cell motility<br>BBB	Glia 63:341-351(2015)<br>Front Cell Neurosci. 2020;14:139.
TSPAN2	tetraspanin 2	6.04 ↑	• Forms large transmembrane protein networks.<br>• Regulate cell motility and invasion.<br>• Regulates cell/ECM adhesion, cell/cell interaction and migration.<br>• Involved in the progression of the lung tumor metastasis.	cell motility<br>cancer	J Cell Biol (2001) 155;(7): 1103<br>Biochem Soc Trans. 2017;45(2):465-475.

NOX4	NADPH oxidase 4	5.99 ↑	• Regulates glycolysis, glucose & fatty acid oxidation in heart.<br>• Overexpression predicts poor prognosis and promotes tumor progression in human colorectal cancer.<br>• Upregulation of NOX4 by TGF-beta is required for its pro-apoptotic activity in hepatocytes.<br>• Inhibition of NOX4/ROS suppresses neuronal and BBB injury	metabolism<br>cancer<br>TGF-β<br>BBB	JCI Insight 2017;2: e96184<br>Oncotarget. 2017;8(20):33586-33600<br>J Hepatol 2008, 49(6):965<br>Front. Cell. Neurosci., 2020, Sect. Cell. Neuropath.doi.org/10.3389/ fncel.2020.578060
ELN	elastin	5.95 ↑	• ECM protein that lends elasticity and resilience to tissues such as the arteries.<br>• TGF-ß is involved in regulation of elastin deposition during fetal development, tissue repair, and in pathological conditions.<br>• In many malignancies, the remodeled ECM expresses high levels of the elastin which may have either positive or negative effects on tumor growth.	cell motility<br>TGF-β<br>cancer	Lab Invest. 1992;66(5):580-8<br>Tumor Microenvironment 2020: Extracellular Matrix Components – Part B: 1
AMIGO2	adhesion molecule with Ig-like domain 2	5.49 ↑	• Controls cell survival and angiogenesis via Akt activation.<br>• Loss of AMIGO2 in ECs led to apoptosis and inhibition of angiogenesis with Akt inactivation.<br>• Extracellular vesicle-derived AMIGO2 stimulates endothelial cell adhesion to cancer cells.<br>• Most upregulated gene in TGFβ-treated human embryonic palatal mesenchymal cells.	angiogenesis<br>cancer<br>metastasis<br>TGF-β<br>EMT?	J Cell Biol (2015) 211 (3): 619.<br>Scientific Reports 2020,12, Art.No.: 792<br>Front Physiol. 2012;3:85.
AC112721.1	uncharacterized	5.30 ↑	• Autophagy-related lncRNA.<br>• Associated with pathways closely related to tumor metastasis and invasion (cytoskeleton regulation, gap junctions, focal adhesion, ECM receptor interaction)	autophagy<br>cell motility<br>cancer	*Front Oncol*. 2021;11:647236. doi:10.3389/fonc.2021.647236
CHRNA9	cholinergic receptor, nicotinic, alpha 9 (neuronal)	5.22 ↑	• Member of the ligand-gated ionic channel family and nicotinic acetylcholine receptor gene superfamily<br>• Involved in ROS regulation.<br>• Important for lung tumor carcinogenesis.	ROS regulation<br>cancer	Int. J. Mol. Sci. 2020, 21(14): 4918<br>J Exp Clin Med 2011, 3(6): 246
PRR5L	proline rich 5 like	4.94 ↑	• modulates mTORC2 activity which is known to regulate glucose uptake, glycolysis and the pentose phosphate pathway (PPP).	metabolism	Nature Cell Biology 2012, 14:686
EGR2	early growth response 2	4.92 ↑	• Regulation of cholesterol/lipid biosynthetic genes<br>• during peripheral nerve myelination.	metabolism<br>senecsence	J Neurochem 2005;93:737<br>Aging Cell. 2021, 20(3):e13318

			• Regulator of senescence.		
MMP-10	matrix metallo-peptidase 10	4.81 ↑	• Upregulated by TGF-ß.<br>• Promotes migration, invasion and angiogenesis.<br>• In the brain, MMPs are critical for BBB integrity.	cell motility<br>angiogenesis<br>TGF-β<br>Cancer<br>BBB (?)	PLoS One 2011, 6(10):e25438<br>Front Oncol. 2019, 9:1370. doi:10.3389/fonc.2019.0137<br>J Cereb Blood Flow Metab. 2016;36(9): 1481
RP11-492E3.2	non coding RNA	4.79↑	• Exosomal antisense ncRNA.		lncRNAfunc: Functional analysis of Long non-coding RNAs - Search (uth.edu)
ADAM19	ADAM metallo-peptidase domain 19	4.53 ↑	• Endopeptidase that cleaves ECM proteins.<br>• Protective biomarker in human prostate cancer.<br>• Target for metabolic syndrome in human and mice.<br>• Regulated by TGF-β.	cell motility<br>cancer<br>metabolism<br>TGF-β	Curr Pharm Des. 2009;15(20):2336<br>BMC Cancer 2016, 16: Art.No. 15<br>Mediators of Inflammation 2017, Art.No.: 7281986
NXPH4	neurexophilin 4	4.47 ↑	• Secreted neuropeptide-like glycoproteins.<br>• Regulates specific synapse function.<br>• Exerts co-receptor function for TGF-ß on the membrane of cancer cells and enhances responses to TGF-ß	cancer<br>TGF- β	eLife 2019;8:e46773<br>Carcinogenesis 2011, 32(4): 613
IL11	interleukin 11	4.43 ↑	• TGF-ß1 stimulates IL11 expression in epithelial cells and fibroblasts.<br>• Stimulates fibrosis which also needs activated pericytes.<br>• Stimulate survival and proliferation of cancer cells alongside angiogenesis of the primary tumor.	cancer<br>TGF- β<br>proliferation<br>angiogenesis	Exp Mol Med 2020, 52: 1871<br>Front. Cell Dev. Biol.2020, 14: 1<br>J Clin Periodontol. 2006, 33(3):165<br>Cytokine & Growth Factor Reviews 2015, 26 (5): 489
RP11-509E16.1	lncRNA	4.33 ↑	• Involved in T cell differentiation.<br>• Triggers the dissemination of colorectal cancer cells via upregulation of Zeb1.	cancer<br>EMT	Nature Comm 2015, 6:6932; doi: 10.1038/ncomms7932<br>Mol Cancer. 2019 Apr 13;18(1):87
SAMD11	sterile alpha motif domain containing 11	4.32 ↑	• Predicted to enable histone binding activity.<br>• Protein domain specific binding activity.<br>• Protein self-association.<br>• Suggested to be involved in cervical cancer.	cancer	SAMD11 Gene - GeneCards<br>Clin Exp Pharmacol Physiol. 2022, 49(1):175
DIO3	deiodinase, iodothyronine, type III	4.24 ↑	• Crucial role in human carcinogenesis.<br>• Role in the regulation of cell proliferation in several neoplastic contexts.<br>• Expression induced by TGF-β.	cancer<br>proliferation<br>TGF-β	Minerva Endocrinol. 2012, 37(4):315-27<br>Oncogene 2021, 40: 6248<br>Molecular Endocrinology 2005, 19(12): 3126
PTHLH	parathyroid hormone-like hormone	4.23 ↑	• Autocrine/paracrine ligand, regulates cell differentiation and proliferation.<br>• Possible role in angiogenesis.<br>• Important in normal and abnormal calcium metabolism.<br>• TGF-ß stimulates PTHLH and osteolytic metastases.	proliferation<br>angiogenesis<br>cancer<br>TGF-β	Entry - *168470 - PARATHYROID HORMONE-LIKE HORMONE; PTHLH - OMIM<br>J Biol Chem 2002, 277(27):24571

BMF	Bcl2 modifying factor	4.16 ↑	• BCL2 protein family.<br>• Binds BCL2 proteins and function as an apoptotic activator.<br>• Might act as a sentinel for stress-impaired protein translation machinery.<br>• Upregulated by TGF-ß.	apoptosis<br>tanslation<br>cancer<br>TGF-β	Cell Death Differ. 2010, 17 (11): 1672.<br>Oncogene 2007, 26(7): 970
SEMA7A	semaphorin 7A	4.15 ↑	• Involvement in migration and differentiation of neurons.<br>• Promotes axon outgrowth.<br>• Neuronal guidance protein.<br>• Promotes chemokine-driven dendritic cell migration.<br>• Plays a critical role in TGF-ß-induced pulmonary fibrosis.<br>• Up-regulated in breast cancer and promotes macrophage production of angiogenic molecules.	cell motility<br>cartilage intermediate layer protein,<br>nucleotide<br>pyrophosphohydrolase<br>cancer<br>angiogenesis	Immunol Res. 2014, 58(1):159<br>Immunol Res. 2013, 57(1-3):81<br>J Exp Med 2007, 204(5): 1083<br>J Immunol 2014, 192 (1 Suppl.) 138.34
AC020571.3	lncRNA	4.11 ↑	• Unknown function.		
LBH	limb bud and heart development	3.94 ↑	• Functions as a Tumor Suppressor of Nasopharyngeal carcinoma by inducing G1/S cell cycle arrest.<br>• Mediates proliferation, fibroblast-to-myofibroblast transition and EMT-like processes in cardiac fibro-blasts.<br>• TGF-ß induces LBH expression in nasopharyngeal epithelial cells.	proliferation<br>cancer<br>EMT	Scientific Rep. 2015, 5, Art.No. 7626<br>Mol Cell Biol 2021, 476(7): doi.org/ 10.1007/s11010-021-04111-7
RP1-27K12.4	Lnc RNA	3.91 ↑	• Unknown function		
ALQX5AP	Arachdonate 5-lipoxygenase-activating protein	3.87 ↑	• Arachidonate binding protein.<br>• Essential for cellular leukotriene (LT) synthesis<br>• Predicts poor prognosis by enhancing M2 macrophage polarization and immunosuppression in ovarian cancer microenvironment.	metabolism<br>cancer	FEBS Lett. 1993, 8, 318(3):277<br>Front. Oncol. 2021,Sec. Gynecological Oncology, doi.org/10.3389/fonc.2021. 675104
CLIP	cartilage intermediate layer protein, nucleotide pyrophosphohydrolase	3.66 ↑	• Matrix protein that resides in the middle of human articular cartilage.<br>• Regulates the extracellular microenvironment of the NVU.<br>• Suppresses TGF-ß signaling in cardiac fibroblasts.	TGF-β	Int J Mol Med 2021, 47(2):475<br>Int J Gerontology 2017, 11(2): 67
PLEK2	Pleckstrin 2	3.61 ↑	• Regulates cell protrusions such as lamellipodia and filopodia.<br>• Involved in tumorigenesiss and metastasis.	cell motility<br>cancer<br>TGF-β	Front. Cell Dev. Biol., 2021<br>Sec. Molecular and Cellular Oncology<br>https://doi.org/10.3389/fcell.2021.768238<br>Cell Death & Disease 2021,12, Art.N0.: 901

			• TGF-β-induced PLEK2 promotes metastasis and chemoresistance in oesophageal squamous cell carci-noma.		
NEDD9	neural precursor cell expressed, developmentally down-regulated 9	3.60 ↑	• Docking protein which plays a central coordinating role in cell adhesion.<br>• Functions in transmitting growth control signals between focal adhesions and the mitotic spindle, this initiating cell proliferation.<br>• Role in integrin beta-1 signaling in B- and T-cells.<br>• TGF-ß target gene.<br>• Promotes oncogenic signaling and a stem/mesen-chymal gene signature in ovarian cancer.	cell motility<br>proliferation<br>TGF-β<br>cancer<br>EMT (?)	Immunity 2006, 25:907<br>PLoS One. 2016;11(6): e0157992.<br>Oncogene 2018, 37: 4854
PPP1R14C	Protein phosphatase 1, regulatory subunit 14C	3.58 ↑	• Modulates capabolic response of osteoblasts.<br>• Upregulated in triple-negative breast cancer.<br>• Might regulate TGF-ß signaling.	metabolism<br>cancer<br>TGF-β	EBioMedicine 9, 2016: 45<br>Biochem J. 2010;430(2):191
FOXQ1	Foxhead box Q1	3.53 ↑	• Regulates including cell proliferation, differentiation and development, especially tumor proliferation.<br>• Overexpression delayed cellular senescence in human fibroblasts.<br>• Mediates the crosstalk between TGF-β and Wnt signaling pathways.	proliferation<br>cancer<br>TGF-β	Cell Death & Disease 2017, 8:2946<br>Cancer Biol Ther 2015;16(7):1099
CDH6	Cadherin 6	3.46 ↑	• Cell adhesion molecule invovled in renal cancer.<br>• Target in TGF-β signalling pathway.	cell motility<br>cancer<br>TGF-β	Cancer Res. 1997, 57(13):2741<br>PLoS One 2013,12;8(9): e75489
RASD1	RAS, dexamethasone-induced 1	3,41 ↑	• Can activate G proteins in a receptor-independent manner and inhibits signal transduction through different G protein-coupled receptors.<br>• Pivotal role in maintaining the equilibrium between adipogenesis and osteogenesis.<br>• Overexpression of RASD1 inhibits glioma cell migration/invasion.	Metabolism<br>cancer<br>cell motility	Metabolism 2020, 108: 154250<br>Scientific Reports 2017, 7, Art.No.: 320
F2RL1	Coagulation factor 2 receptor like 1	3,25↑	• Promotes TGF-ß dependent cell motility in panceatic cancer cells.	cell motility<br>cancer<br>TGF-β	Oncotarget. 2016, 7(27):41095
SYTL5	synaptotagmin-like 5	3.22 ↑	• Rab effector protein.<br>• Binds phospholipids in neuroendocrine cells.<br>• Regulates exocytosis.<br>• Involved in EMT processes in NSCLC.	cancer<br>EMT<br>proliferation<br>cell motility	NextProt, Madame Curie Bioscience Database<br>EBioMedicine 2019, 46: 42-53

			• Promotes proliferation, invasion, and metastasis and inhibited cell apoptosis of NSCLC cells.		
DHRS2	dehydrogenase/reductase (SDR family) member 2	3.22 ↑	• Mitochondrial gene.<br>• Involved in energy generation processes.<br>• Closely associated with the inhibition of cell proliferation, migration and quiescence in cancers.	metabolism<br>cancer<br>proliferation<br>cell motility	UniProt<br>J Exp Clin Cancer Res 2019, 38, Art. No.: 30
IFIH1	Inferferon induced with helicase C domain 1	3,20 ↑	• Involved in virus-induced autoimmune diabetis type II	metabolism	Arq Bras Endocrinol Metabol 2013, 57(9):667
WNT5B	wingless-type MMTV integration site family, member 5B	3.16 ↑	• Supresses Smad2/3 activation.<br>• Regulates canonical and non-canonical Wnt-pathway during adipositas and diabetes type II.	metabolism<br>TGF-β	J Diab Invest 2020, 11(2): 307<br>Signal Transduction 2010, 285 (18): 14031<br>J Diabetes Investig 2020,11(2): 307
MGP	matrix Gla protein	3.14 ↑	• Highly expressed by vascular smooth muscle cells (VSMCs).<br>• Lack of MGP causes vascular abnormalities.<br>• Might contribute to breast cancer resistance mechanism by augmenting the interaction of cells with ECM components.	Angiogenesis<br>cell motility (?)	Curr Med Chem 2020, 27(10):1647<br>Angiogenesis 2016, 19: 1<br>Int. J. Mol. Sci. 2018, 19: 2901
ZNF365	Zinc finger protein 365	3.13 ↑	• Maternal LPS-binding protein.<br>• Downregulation associated with poor prognosis in colon cancer.<br>• Overexpressed in TGF-ß mediated pulmonary fibrosis	Cancer<br>TGF-β	FASEB 2018, 31 (2): 979<br>Oncol Lett. 2020;20(4):85<br>FASEB 2020, 34 (S1, Exp. Biology): 1
CLDN14	Claudin 14	3.08 ↑	• Extracellular calcium metabolism<br>• Cell adhesion molecule, upregulated in gastric cancer.<br>• Part of endothelial tight junctions	cell motility<br>proliferation<br>cancer<br>BBB (?)	*JASN* 2014,25 (4) 745<br>Diagnostic Pathology 2013, 8: Art. No.: 205<br>Tissue Barriers. 2013;1(3): e24782
HAS2	hyaluronan synthase 2	3.07 ↑	• Responsible for the synthesis and deposition of hyaluronan in the ECM.<br>• Overexpression of HAS2 lin renal epithelial tip cells led to enhanced cell migration.<br>• Silencing of HAS2 suppresses the malignant phenotype of invasive breast cancer cells.<br>• TGFβ-induced EMT depends on hyaluronan synthase HAS2.	cell motility<br>cancer<br>EMT<br>TGF-β	JASN June 2006, 17 (6):1553<br>Int J Cancer 2007, 120(12): 2557<br>Oncogene 2013,32(37): 435
KCNN4	K^+ intermediate /small conductance calcium-activated channel, subfamily N, member 4	3.06 ↑	• Forms a voltage-independent potassium channel<br>• Promotes invasion and metastasis through MAPK/ERK.<br>• Targeted inhibition of KCa3.1 attenuates TGF-β-induced reactive astrogliosis.	cell motility<br>TGF-β	J Investig Med. 2020, 68(1):68<br>J Neurochem 2014, 130(1): 41

HECW2	HECT, C2 and WW domain containing E3 ubiquitin protein ligase 2	3.06 ↑	• Ubiquitin ligase.<br>• Stabilises p73<br>• promotes endothelial cell junctions	BBB (?)	J Med Genet. 2017;54(2):84<br>Cellular Signalling 2016, 28: 1642
SLC46A3	solute carrier family 46, member 3	3.05 ↑	• Involved in plasma membrane electron transport.<br>• Plasma membrane analog of the mitochondrial electron transport chain.	metabolism	Current Molecular Med 2006. **6** (8): 895
GPR68	G protein-coupled receptor 68	2.95 ↑	• Involved in pH homeostasis.<br>• Modulates intestinal inflammation and fibrosis.<br>• Mediates the interaction of cancer-associated fibroblasts and cancer cells<br>• Expression regulated by TGF-ß<br>• Indirectly influences glycolysis	cancer<br>TGF-β<br>metabolism	Inflamm Intest Dis 2021;6:140<br>FASEB J. 2018, 32(3):1170<br>J Immunol May 1, 2020, 204 (1 Supplement) 76.22<br>BBA - Molecular Basis of Disease 2019, 1865 (12): 165537
STK38L	serine/threonine kinase 38 like	2.87 ↑	• Ablation promotes loss of cell viability in a subset of KRAS-dependent PCC.	cancer	Oncotarget 2017,8(45):78556
PMEPA1	prostate transmembrane protein, androgen induced 1	2,83 ↑	• Suppresses the aTGF-ß signaling pathways though interactions with Smad proteins.<br>• Overexpression of this gene may play a role in multiple types of cancer.	TGF-β<br>proliferation<br>cell motility<br>cancer	PMEPA1 prostate transmembrane protein, androgen induced 1 [Homo sapiens (human)] - Gene - NCBI (nih.gov)
ITGB3/CD61	integrin, beta 3 (platelet glycoprotein IIIa, antigen CD61)	2.83 ↑	• Associated with the risk of human cancers.<br>• Key regulator in reactive oxygen species-induced migration and invasion of CRC.<br>• In breast cancer cells enhances TGF-β signaling.<br>• Facilitates EMT and angiogenesis.	cancer<br>cell motility<br>TGF-β<br>EMT<br>angiogenesis	Mol Cell Proteomics. 2011;10(10): M110.005397<br>Oncotarget. 2017;8(70):114856<br>Am J Transl Res. 2019;11(12):7195
RGS9	regulator of G-protein signaling 9	2,82 ↑	• Increases GTPase activity of G protein alpha subunits.		RGS9 regulator of G protein signaling 9 [Homo sapiens (human)] - Gene - NCBI (nih.gov)
SLC19A2	solute carrier family 19 (thiamine transporter), member 2	2.80 ↑	• Thiamin transporter.<br>• Upregulation in stem cell like cancer cells of NPCC contributes to enhanced gylcolysis.	cancer (?)<br>metabolism	Int J Radiation Oncoll Bio Physics 2016,96 (2) Suppl. E582
SKIL	SKI-like oncogene	2.77 ↑	• component of the SMAD pathway, which regulates cell growth and differentiation through TGF-ß<br>• involved in EMT processes.	TGF-β<br>cancer<br>EMT	SKIL SKI like proto-oncogene [Homo sapiens (human)] - Gene - NCBI (nih.gov)<br>Cell Death Differ 2021,28(1):267
MFSD7	major facilitator superfamily domain containing 7	2.77 ↑	• Predicted to enable transmembrane transporter activity.<br>• Predicted to be involved in transmembrane transport.		SLC49A3 Gene - GeneCard
MYO1D	myosin ID	2.75 ↑	• Evolutionarily conserved regulator of animal left–right asymmetry.	cancer<br>TGF-β	Nat Commun. 2018;9(1):1942.<br>Oncogene 2019, 38:7416<br>Int J Mol Sci. 2019;20(16):3913.

			• Overexpressed MYO1D promotes colorectal tumor progression through upregulating the EGFR in the plasma membrane.<br>• Might regulate TGF-ß receptor trafficking		
NEBL	nebulette	2.74 ↑	• cardiac-specific isoform of nebulin family proteins.<br>• Regulates stability and length of actin thin filaments.<br>• New susceptibility gene for endocardial fibroelastosis.	vessel dilatation (?)	J Am Coll Cardiol. 2010, 56 (18) 1493
PRG4	proteoglycan 4	2.73 ↑	• Codes for lubricin.<br>• Protects cartilage surface from protein deposition and cell adhesion.<br>• Close analog to vitronectin.<br>• Putative role in cell-cell and cell-ECM interactions.<br>• TGF-ß regulates PRG expression in vascular smooth muscle cells.<br>• Involved in tumor angiogenesis.	cell motility (?)<br>TGF-β<br>angiogenesis	J Diabetes 2010, 2(4):233<br>Semin Cancer Biol. 2020;62:1
HEY1	hes-related family bHLH transcription factor with YRPW motif 1	2.73 ↑	• Basic HLH transcription factor, inhibits myogensis<br>• Induces cell proliferation in neural stem cells<br>• Upregulated in glioma.<br>• Promotes migration& invasion of melanoma cells	cancer<br>proliferation<br>cell motility	J Cell Mol Med 2009,13(1):136<br>J Cancer. 2021;12(23):6979
CLDN4	claudin 4	2.72 ↑	• silencing promotes proliferation of gastric cancer cells through activation of PI3K/Akt.<br>• in GBM TGF-ß mediated upregulation of CLDN4 induces EMT.<br>• tight junction protein, expressed in endothelial cells.	proliferation<br>cancer<br>cell motility<br>EMT<br>TGF-β<br>BBB	Exp Physiol 2020,105(6):979<br>Cell Death & Disease 2022,13, Art.No.: 339<br>Neoplasia 2012,14: 974
AC002454.1	LncRNA	2.70 ↑	• AC002454.1 and CDK6 synergistically promote endometrial cell migration and invasion in endometriosis.	cell motility	Reproduction 2019,157(6):535
WNT2	wingless-type MMTV integration site family member 2	2.70 ↑	• Autocrine WNT2 signaling in fibroblasts promotes colorectal cancer progression.<br>• Cooperates with TGF-ß signaling.<br>• Increases tumor angiogenesis in colon cancer.	Cancer<br>TGF-β<br>angiogenesis	Oncogene 2017, 36: 5460<br>Angiogenesis 2020, 23: 159
REPS2	RALBP1 associated Eps domain containing 2	2.64 ↑	• Regulates the endocytosis of growth factor receptors (downregulated in cancer cells)	cancer	Asian Pac J Cancer Prev 2013, 14(5):2851
TPM1	tropomyosin 1 (alpha)	2.63 ↑	• Functions as a tumor suppressor (proliferation, angiogenesis &, metastasis) in renal cell carcinoma.<br>• Expression regulated by TGF-ß.<br>• Cell elasticity is regulated by the tropomyosin isoform composition of the actin cytoskeleton.	cancer<br>proliferation<br>cell motility	J Cancer. 2019;10(10): 2220<br>J Cell Mol Med 2017, 21(5): 916<br>PlosOne 2015, doi.org/10.1371/ journal.pone.0126214

PPP1R13L	protein phosphatase 1, regulatory subunit 13 like	2.63 ↑	• Encodes the inhibitor of apoptosis-stimulating protein of p53 protein (iASPP).<br>• Causes paediatric dilated cardiomyopathy.<br>• Acts as a regulator of desmosomes.<br>• Has been implicated in inflammatory pathways.<br>• iASPP is essential for HIF-1alpha stabilization to promote angiogenesis and glycolysis via attenuating VHL-mediated protein degradation.<br>• Downregulation of iASPP expression suppresses proliferation and invasion of HNSCC.	angiogenesis<br>metabolism<br>cancer<br>cell motility<br>proliferation	Clin Genet 2020, 98(4):331<br>Oncogene 2022, 41: 1944<br>Chin Med Sci J 2019, 34(3):184
STK17B	serine/threonine kinase 17b	2.63 ↑	• promotes carcinogenesis and metastasis via AKT/GSK-3β/SNAIL signaling in HCC.	cancer<br>EMT<br>cell motility	Cell Death & Disease 2018, **9**, Art.No.: 236
CDH2	cadherin 2, type 1, N-cadherin (neuronal)	2.62 ↑	• E-Cadherin to N-Cadherin switch in EMT.	EMT<br>cell motility<br>cancer	Cells. 2019;8(10):1118.
CASC15	cancer susceptibility candidate 15 (non-protein coding)	2.61 ↑	• Tumor-Associated Long Non-Coding RNA<br>• Can affect proliferation, invasion and apoptosis of tumors	proliferation<br>cancer<br>cell motility	Curr Pharm Des 2021, 27(1):127
ANKRD44	ankyrin repeat domain 44	2.61 ↑	• Putative regulatory subunit of PP6 that may be involved in the recognition of phosphoprotein substrates.		ANKRD44 - Serine/threonine-protein phosphatase 6 regulatory ankyrin repeat subunit B - Homo sapiens (Human) | UniProtKB | UniProt
ADAMTS6	ADAM metallopeptidase with thrombospondin type 1 motif, 6	2.60 ↑	• Suppresses tumor progression via the ERK signaling pathway.<br>• Differentially expressed in brain pericytes, putatively associated to BBB density.	cancer<br>BBB (?)	Oncotarget 2016,7(38):61273<br>Cells 2021, 10, 963, doi.org/10. 3390/cells10040963
PDGFA	platelet-derived growth factor alpha polypeptide	2.58 ↑	• Activator of the PDGFR-beta who´s expression is upregulated in activated pericytes.<br>• essential and autocrine regulator of VEGFR ex-pression in NSCLC.<br>• regulation of cell motility during wound healing.	cancer<br>angiogenesis<br>cell motility	Neuropathol Appl Neurobiol 2021, 47(6):768<br>Cancer Res 2005,65(16):7241<br>Cell Physiol Biochem 2010, 25(2-3):279
INSC	inscuteable homolog (Drosophila)	2.58 ↑	• Involved in spindle orientation during mitosis.<br>• May regulate cell proliferation and differentiation in the developing nervous system.	Proliferation (?)	INSC - Protein inscuteable homolog - Homo sapiens (Human) | UniProtKB | UniProt
CRLF1	cytokine receptor-like factor 1	2.58 ↑	• may have an ability to change the composition of ECM in fibrosis.<br>• positively regulated by TGF-β in the mouse chondrogenic cells.	cell motility (?)<br>TGF-β<br>proliferation	World J Hepatol. 2012;4(12):356<br>Calcified Tissue Int 2010, 86:47
DNAJB5/Hsc40	DnaJ (Hsp40) homolog, subfamily B, member 5	2.56 ↑	• Involved in the regulation of ROS	cancer	Antioxid Redox Signal. 2013;18(10):1165

NUAK1	NUAK family, SNF1-like kinase, 1	2,52 ↑	• Phosphorylates p53 and regulates proliferation.<br>• Cytosolic NUAK1 enhances ATP production by maintaining proper glycolysis and mitochondrial function in cancer cells.<br>• Expression induced by TGF-ß.	proliferation<br>cancer<br>metabolism<br>TGF-β	Oncogene 2011,30(26):2933<br>Front. Oncol., 2020, Sec. Cancer Metabolism, doi.org/10.3389/fonc.2020.01123<br>J Biol Chem 2019,15;294(11):4119
TNFSF11	tumor necrosis factor (ligand) superfamily, member 11	2.51 ↑	• Cytokine that binds to TNFRSF11B/OPG and to TNFRSF11A/RANK.<br>• Osteoclast differentiation and activation factor.		TNFSF11 - Tumor necrosis factor ligand superfamily member 11 - Homo sapiens (Human) | UniProtKB | UniProt
EDN1	endothelin 1	2.51 ↑	• Potent vasoconstrictor peptide produced by vascular endothelial cells.<br>• Elevated plasma concentration in several solid tumors, multiple functions in cancer cells.	cancer<br>proliferation<br>angiogenesis<br>cell motility	Pharmacolog Rev 2016, 68 (2): 357<br>Br J Cancer. 2003;88(2):163.
BPGM	2,3-bisphosphoglycerate mutase	2.50 ↑	• central part of the Rapoport-Luebering-cycle, a side pathway of glycolysis.<br>• controls the serine pathway flux.	metabolism	Nat Chem Biol 2017,13(10):1081
VGLL3	vestigial like 3 (Drosophila)	2.50 ↑	• inhibitor of adipocyte differentiation.<br>• regulates cranial neural crest migration.	cell motility<br>differentiation	J Lipid Res 2013, 54(2):473<br>Biol Open. 2017;6(10):1528<br>J Cell Mol Med 2022, 26 (9):2686
F3	coagulation factor III (thromboplastin, tissue factor)	2.47 ↑	• Enables cells to initiate the blood coagulation cascades.<br>• Participates in many tumour-related processes that contribute to malignant disease progression	cancer<br>angiogenesis<br>BBB	Breast Cancer Res. 2008;10(2):204
SMAD7	SMAD family member 7	2.47 ↑	• Negatively regulates TGF-ß signaling	TGF-β<br>cancer	Acta Biochim Biophys Sin 2009;41(4):263
BHLHE40	basic helix-loop-helix family, member e40	2.46 ↑	• confers pro-survival and pro-metastatic phenotype to breast cancer.<br>• overexpression in multiple cell types has been shown to inhibit cell proliferation, migration, or invasion.<br>• Regulates TWIST transcription in cncer cells.	proliferation<br>cell motility<br>EMT<br>cancer	Breast Cancer Res 2018, 20, Art.No.: 117<br>Mol Cellular Biol 2015, 35(24): 4096
DIXDC1	DIX domain containing 1	2.46 ↑	• Regulate β-catenin stability.<br>• Regulates cell stickyness.	cell motiliy<br>cancer	Mol Cell Biol. 1999;19(6):4414<br>medicalxpress.com
DGKI	diacylglycerol kinase, iota	2.45 ↑	• regulates intracellular concentration of diacylglycerol<br>• can mediate growth factors to induce mitosis<br>• overexpressed in cancer	cancer<br>proliferation	J Biol Chem. (1998) 273:32746<br>Biochim Biophys Acta. (2000) 1483:199<br>Front. Med., 2020, Sec. Gastroenterology doi.org/10.3389/fmed.2020.00320
PODXL	podocalyxin-like	2.42 ↑	• Might be a new prognostic biomarker in various cancers.<br>• In B cell lymphoma promotes cell proliferation, survival, migration, resistance.	cancer<br>proliferaton<br>cell motility<br>EMT	BMC Cancer 2020, 20, Art.No.: 620<br>Cancers 2020;12(2):396<br>PLoS One 2011,12;6(4):e18715

			• Requirement of podocalyxin in TGF-beta induced epithelial mesenchymal transition.	TGF-β	
LTBP2	latent transforming growth factor beta binding protein 2	2.41 ↑	• a major component of arterial tissue.<br>• Modulator of TGF-ß activity.<br>• Promotes the migration and invasion of gastric cancer cells<br>• As an angiogenic factor, LTBP2 upregulation elevated the tube formation of HUVECs	TGF-β<br>cancer<br>cell motility<br>angiogenesis	Int J Oncol. 2018;52(6):1886<br>Mol Cell Endocrinology, 2022, 550: 111647,
TNS1	tensin 1	2.41 ↑	• localizes to focal adhesions.<br>• may promote EMT and cancer cell metastasis.<br>• Plays an essential role in TGF-β-induced myofibroblast differentiation and myofibroblast-mediated formation of extracellular fibronectin and collagen.	EMT<br>Cancer<br>TGF-β<br>cell motility (?)	Arch Med Sci 2020, doi.org/10.5114/aoms/127085<br>Am J Respir Cell Mol Biol 2017, 56(4):465
RASGRP3	RAS guanyl releasing protein 3 (calcium and DAG-regulated)	2.38 ↑	• Controls cell proliferation and migration in cancer	cancer<br>cell motility	Gene 2017,633:35<br>Clin Exp Pharmacol Physiol 2018,45(7):720
XYLT1	xylosyltransferase I	2.38 ↑	• XYLT1 is actively regulated by TGF-β.<br>• Initiates glycosaminoglycan synthesis	metabolism (?)<br>TGF-β	Thesis: The regulation of XYLT1, the enzyme initiating glycosaminoglycan synthesis; An in vitro study with human immortalized fibroblasts with integrated reporter plasmid (uni-muenchen.de)
JUNB	jun B proto-oncogene	2.35 ↑	• governs a feed-forward network of TGF-ß signaling that aggravates breast cancer invasion.<br>• Stimulates proliferation.	cell motiliy<br>cancer<br>TGF-β<br>proliferation	JunB proto-oncogene-NCBI (nih.gov)<br>The EMBO Journal (2002)21:4104
NEK7	NIMA-related kinase 7	2.34 ↑	• Regulation of mitosis<br>• activation of the NLRP3 inflammasome	proliferation<br>inflammation	Genome Res. 2003, 13: 1366<br>Nat. Immunol. 2016, 17: 250
GPR115	G protein-coupled receptor 115	2. 33 ↑	• Contributes to lung adenocarcinoma metastasis.<br>• Involved in processes of proliferation, migration, invasion and angiogenesis	cancer<br>proliferation<br>cell motility<br>angiogenesis	Front. Oncol., 2020, Sec. Molecular and Cellular Oncology doi.org/10.3389/ fonc.2020.577530
TGFBR1	transforming growth factor, beta receptor 1	2.29 ↑	• Receptor for TGF-ß.<br>• If activated induces EMT, cell motility and proliferation of pericytes.<br>• Controls angiogenesis by two unique TGFBR1 signaling pathways.	cell motility<br>TGF-β<br>proliferation<br>EMT<br>Cancer<br>angiogenesis	Neuropathol Appl Neurobiol 2021, 47(6):768<br>Histol Histopathol 2011,26(9):1219
TMEM2	transmembrane protein 2	2.24 ↑	• Expression induced by TGF-ß.<br>• Hyaluronidase<br>• Predicts poor prognosis in PDAC.	TGF-β<br>cancer<br>cell motility	Biochem Biophys Res Commun 2018, 505(1):74<br>Pancreatology 2020,20(7):1479

			• Regulates cell adhesion and migration via degradation of hyaluronan at focal adhesion sites.		J Biol Chem 2021,296:100481
SRRM3	serine/arginine repetitive matrix 3	2.24 ↑	• RNA splicing factor.<br>• Associated with cancer.	cancer	Cancer Res 2021,81(18):4736
SYN1	synapsin I	2.22 ↑	• Mutations may be associated with X-linked disorders with primary neuronal degeneration	neuronal development	BMC Med Genomics 2021,14(1):182
WNT9A	wingless-type MMTV integration site family, member 9A	2.21 ↑	• Induction linked to suppression of human colorectal cancer cell prolferation.	Cancer<br>proliferation	Int J Mol Sci. 2016;17(4):495.
TSPAN13	tetraspanin 13	2.20 ↑	• modulates voltage-gated Cav2.2 Ca^{2+} channels.<br>• play a role in the regulation of cell development, activation, growth and motility.<br>• tumor suppressor.	cell motiliy<br>cancer<br>proliferation	Scientific Reports 2013, 3, Art.No.: 1777<br>Bosn J Basic Med Sci. ,19(2):146
GREM1	gremlin 1, DAN family BMP antagonist	2.19 ↑	• inhibitor in the TGF-ß signaling pathway.<br>• expression is found in many cancers.<br>• plays a BMP-dependent role.<br>• in angiogenesis on endothelium of human lung tissue.	TGF-β<br>cancer<br>angiogensis	BMC Cancer 2006, 6: 74<br>Blood 2007, 109 (5): 1834
SNAI1	snail family zinc finger 1	2.18 ↑	• encodes the SLUG protein<br>• induction of EM in human brain pericytes<br>• associated with enhanced migration and proliferation of brain pericyes	cell motiliy<br>cancer<br>proliferation<br>TGF-β	Neuropathol Appl Neurobiol 2021,47(6): 768
VDR	vitamin D (1,25-dihydroxyvitamin D3) receptor	2.18 ↑	• VDR signaling enhances adhesion and suppresses the invasive capacity of cells.<br>• loss of VDR can lead to abnormal tumor angiogenesis.<br>• Required for proliferation, migration, and differentiation of epidermal stem cells and progeny during cutaneous wound repair.<br>• Negative regulator of TGF-β/Smad signalling in systemic sclerosis.	cell motility<br>angiogeneis<br>cancer<br>proliferation	Endocrinol Metab Clin North Am. 2017;46(4):1009<br>Cancer Res. 2009;69(3):967<br>J Invest Dermatol 2018,138(11):2423<br>Ann Rheum Dis 2015,74(3):e20
VAT1L	vesicle amine transport 1-like	2.17 ↑	• Function unknown		
SPDL1	spindle apparatus coiled-coil protein 1	2.14 ↑	• Functions in mitotic spindle formation	proliferation<br>cancer	Front. Genet 2022, 18 Sec. Compl. Gen. doi.org/10.3389/fgene.2022.79802
ARHGEF40	Rho guanine nucleotide exchange factor (GEF) 40	2.11 ↑	• Rho family GTPases control numerous cellular processes including cytoskeletal reorganization	cell motility (?)	JBC - Mech Signal Transduction 2001, 276(7): 4948
LRRC17	leucine rich repeat containing 17	2.10 ↑	• Anti-apoptotic protein linked to prognosis of ovarian cancer.	cancer	Anticancer Res 2020,40(10): 5601
SLITRK6	SLIT and NTRK-like family, member 6	2.10 ↑	• moderately negatively correlated with tumor malignancy.	cancer	Biochem Biophys Rep 2021, 28: 101157

IL21R	interleukin 21 receptor	2.09 ↑	• Transduces the growth promoting signal of IL21.<br>• Controverse functions in cancer<br>• Modulates functions of T, B, natural killer (NK), and myeloid cells.	cancer<br>immune surveillance	Clin Cancer Res 2007, 13 (23): 6926<br>Blood (2007) 109 (10): 4135
RP11-64D22.2	pseudogene	2.09 ↑	• Promotes growth and tumorigenesis of breast cancer	cancer	Molecular Therapy: Nucleic Acids 2020, 21: 916
TMC7	transmembrane channel-like 7	2.08 ↑	• Predicted to enable mechanosensitive ion channel activity. Predicted to be involved in ion transmembrane transport		TMC7 transmembrane channel like 7 [Homo sapiens (human)] - Gene - NCBI (nih.gov)
SPSB1	splA/ryanodine receptor domain and SOCS box containing 1	2.08 ↑	• Involved in protein ubiquitination and ubiquitin-dependent protein catabolic process<br>• Negative regulator of TGFBR2	TGF-β<br>cancer	J Biol Chem 2015, 290(29):17894
ARHGAP31	Rho GTPase activating protein 31	2.07 ↑	• Negative regulator of Rho GTPase	cancer	J Hematol Oncol 2021,14, Art.No.: 171
DAAM1	dishevelled associated activator of morphogenesis 1	2.06 ↑	• Promotes new actin filaments, regulates cell growth through the stabilization of microtubules.<br>• Overexpression correlates with metastasis in breast cancer.<br>• Activation of DAAM1 leads to inhibition of endothelial cell proliferation, migration, and angiogenesis.	cancer<br>cell motility<br>angiogenesis	Pathol Res Pract 2020, 216(3):152736<br>PNAS 2010;107(15):6906
SPOCK1	sparc/osteonectin, cwcv and kazal-like domains proteoglycan (testican) 1	2.05 ↑	• Extracellular proteoglycan.<br>• Induces EMT in several cancer cells lines<br>• Promotes migration, degradation of the basement membrane, invasion, adhesion and angiogenesis.<br>• Regulates BBB integrity.	cancer<br>EMT<br>TGF- β<br>cell motility<br>proliferation<br>angiogenesis<br>BBB	Biochem. Biophys. Res. Commun. 2013, 440: 792<br>Molecular Cancer 2015,14, Art.No.: 12<br>Cell Physiol 2022, 322 (4): C688
PCDH9	protocadherin 9	2.05 ↑	• acts as a tumor suppressor inducing arrest at G_0/G_1, is frequently methylated in HCC<br>• inhibits EMT in cancer	cancer<br>EMT<br>cell motility	Mol Med Rep. 2017;16(4):4475<br>Cell Death & Disease 2018, 9, Art. No.: 27
MB21D2	Mab-21 domain containing 2	2.04 ↑	• Promotes a pro-oncogenic phenotype in HNSCC.	cancer<br>proliferation	Mol Oncol 2020, 14(12):306
RAP1GAP2	RAP1 GTPase activating protein 2	2.02 ↑	• GTPase-activating protein that activates the small guanine-nucleotide-binding protein Rap1 in thrombocytes.<br>• Inhibits cytoskeletal remodeling and motility in thyroid cancer cells.	cancer<br>cell motility	Endocr Relat Cancer. 2012;19(4):575
HS3ST3A1	heparan sulfate (glucosamine) 3-O-sulfotransferase 3A1	2.01 ↑	• novel tumor regulator and a prognostic marker in breast cancer.	cancer	Oncogene, 2016 Sep 22. PMID 27041583

KIAA1549L	KIAA1549-like	2.01 ↑	• Predicted to be integral component of membrane		KIAA1549 like [Homo sapiens (human)] - Gene - NCBI (nih.gov)
