# Supplementary material for: TGF-Beta Modulates the Integrity of the Blood Brain Barrier In Vitro, and Is Associated with Metabolic Alterations in Pericytes
Source: Biomedicines. 2023 Jan 14;11(1):214. doi: 10.3390/biomedicines11010214 (PMC9855966; doi:10.3390/biomedicines11010214)
Supplement: Supplementary file 1 [file biomedicines-11-00214-s001.zip › Supplementary Table 2.pdf]

Supplementary Table S2: Downregulated genes with a log fold change of >2 in TGF-β treated HBVP

Symbol	Gene Name	Log fold change	Function	Connected to	Reference
RP11-554I8.2		3.71 ↓	Upregulated in bladder cancer		Cell Physiol Biochem 2018;50:1154
PPL	periplakin	3.26 ↓	Adhesion molecule.<br>Links cytoskeletal elements to each other and to the plasma membran.<br>Involved in cellular movement and attachment in cancer.	cell motility	BMC Cell Bio 2011, 12:41
PPARGC1A	peroxisome proliferator-activated receptor gamma, coactivator 1 alpha	3.26 ↓	Transcriptional coactivator of the genes involved in energy metabolism.<br>Master regulator of mitochondrial biogenesis..<br>Pushes muscle fibers to be metabolically more oxidative and less glycolyti.c<br>Participates in the regulation of both carbohydrate and lipid metabolism.	metabolism	Physiol Rep 2006, 30 (4): 145
IL-1B	interleukin 1, beta	3.24 ↓	Important role in lipid metabolism<br>Central role in coordinating host immune and proinflammatory responses	metabolism	J Exp Med 2003, 198 (6): 877<br>Brain Res Rev. 2009;60(1): 57
SPRY1	sprouty homolog 1, antagonist of FGF signaling	2.60 ↓	Membrane-anchored phosphoprotein inhibitors of growth factor signaling in endothelial cells.<br>Molecular control of endothelial cell behaviour during blood vessel morphogenesis	angiogenesis	J Cell Bio 2001,152(5):1087<br>Nat Rev Mol Cell Biol. 2011;12(9):551
CTC-529P8.1		2.79 ↓	Suggested to be involved in angiogenic processes	angiogenesis	http://angiogenes.uni-frankfurt.de/
RP11-215G15.5	lnc RNA	2.71 ↓	Unknown function		
TCF21	transcription factor 21	2.64 ↓	Involved in pathogensis of fibrosis		Am J Pathol 2020, 190(1): 145
SDPR	Serum derivation response	2.61 ↓	Required for blood vessel formation (angiogenesis)<br>Required for endothelial cell proliferation, migration and invasion	angiogenesis<br>cell motility<br>proliferation	J Biol Chem 2017,292 (43): 17760
ZBED3	zinc finger, BED-type conaining 3	2.55 ↓	Axin-binding protein that is involved in Wnt/β-catenin signaling modulation		JBC 2009, 284, 11: 668
ST3GAL5	ST3 beta-galactosidas alpha-2,3-sialyltransferase	2.54 ↓	Downregulation associated with bladder cancer muscle invasion	cell motility	Oncol Let 2020, 20(1): 828
AFAP1L2	actin filament associated proein1 like 2	2.50↓	Adaptor protein involved in many cellular functions, such as cell survival, cell proliferation, migration, and gene and miRNA expression	cell motiliy<br>proliferaton	Scientifica 2014;2014:903014.
IFIT1	interferon-induced protein with tetratricopeptide repeats 1	2.44 ↓	Involved in immune response and antiviral defense.	cell motility	Cancer Res. 2017, 77 (Suppl. 13):3933.

			overexpression<br>of IFIT1 enhanced EGFR and AKT activation and subsequently promoted OSCC invasion through EMT		
THBD	thrombomodulin	2.36 ↓	transmembrane glycoprotein located on the luminal surface of endothelial cells in most normal blood vessels.<br>Regulates coagulation and inflammation.<br>Loss of TM is a prominent feature of postradiation endothelial dysfunction	angiogenesis (?)	Marjan Boerma, in Endothelial Signaling in Vascular Dysfunction and Disease, 2021
NOV	nephroblastoma overexpressed	2.34 ↓	Member of the CCN family of proteins.<br>Expression reagulted by TGF-ß.<br>Provides BMP antagonistic properties		Ann N Y Acad Sci 2007, 1116:50
CXADR	coxsackie virus and adenovirus receptor	2.31 ↓	Component of the epithelial apical junction complex.<br>Function as a cell adhesion molecule.<br>Essential for tight junction integrity.	angiogenesis (?)	PNAS 2001, 98(26): 15191
RP11-38P22.2	Lnc rNA	2.25 ↓	Suggested to be involved in angiogenic processes	angiogenesis	http://angiogenes.uni-frankfurt.de/
GABRB1	gamma-aminobutyric acid (GABA) A receptor, beta 1	2.20 ↓	Inhibitory neurotransmitter for the central nervous system.		GABRB1 gamma-aminobutyric acid type A receptor subunit beta1 [Homo sapiens (human)] - Gene - NCBI (nih.gov)
STAR	Steroidogenic acute regulatory protein	2.06 ↓	promote the movement of cholesterol from the outer to the inner mitochondrial membrane	metabolism	PNAS 1996;93(24):13731
LINC00341	long intergenic non-protein coding RNA 341	2.05 ↓	Exerts anti-inflammatory effect on endothelial cells by repressing VCAM1		Physiol Genomics 2017, 49(7): 339
TNFRSF1B	tumor necrosis factor receptor superfamily, member 1B	2.05 ↓	Associated with diabetes type 2 kidney disease.<br>critical for the promotion of tumor growth, invasion and metastasis.	cell motility	TNFRSF1B TNF receptor superfamily member 1B [Homo sapiens (human)] - Gene - NCBI (nih.gov)<br>PlosOne 2014<br>doi.org/10.1371/journal.pone.0101138
ARHGAP29	Rho GTPase activating protein 29	2.00 ↓	Prognostic factor of proliferation and invasion in prostate cancer.	cell motility<br>proliferatoin	Oncol. Rep. 2020, 44 (6): 2735
