# Supplementary material for: TGF-Beta Modulates the Integrity of the Blood Brain Barrier In Vitro, and Is Associated with Metabolic Alterations in Pericytes
Source: Biomedicines. 2023 Jan 14;11(1):214. doi: 10.3390/biomedicines11010214 (PMC9855966; doi:10.3390/biomedicines11010214)
Supplement: Supplementary file 1 [file biomedicines-11-00214-s001.zip › SupplementaryTable_3.pdf]

Supplementary Table S3: Joint-pathway analysis of metabolomics and RNA sequencing datasets. Most significantly changed pathways of TGF-β vs. control, based on joint-pathway analysis by metabolite concentrations and gene expression fold change, top 14 metabolic pathways.

Pathway	Total	Hits	Raw p	-log10(p)	Holm adjust	FDR	Impact
Glycerolipid metabolism	35	12	1.03E-05	4.9891	0.00086144	0.00086144	0.76471
Valine, leucine and isoleucine biosynthesis	12	6	0.000171	3.7665	0.014209	0.0071902	1.3636
Glycerophospholipid metabolism	86	16	0.001258	2.9004	0.10314	0.030207	0.48235
Amino sugar and nucleotide sugar metabolism	79	15	0.001438	2.8421	0.11651	0.030207	0.61538
Aminoacyl-tRNA biosynthesis	74	14	0.002146	2.6684	0.17165	0.034949	0.19178
Ascorbate and aldarate metabolism	13	5	0.002626	2.5807	0.20743	0.034949	0.75
Glycolysis or Gluconeogenesis	61	12	0.003155	2.501	0.24609	0.034949	0.75
Histidine metabolism	32	8	0.003329	2.4778	0.25629	0.034949	0.45161
Alanine, aspartate and glutamate metabolism	61	11	0.009206	2.0359	0.69965	0.085922	0.65
Glycosaminoglycan biosynthesis - chondroitin sulfate / dermatan sulfate	18	5	0.012458	1.9046	0.93435	0.10465	0.70588
Glutathione metabolism	56	10	0.013718	1.8627	1	0.10476	0.8
Phosphatidylinositol signaling system	74	12	0.015369	1.8133	1	0.10759	0.47945
Glycine, serine and threonine metabolism	68	11	0.020321	1.6921	1	0.1313	0.71642
Phenylalanine metabolism	21	5	0.024189	1.6164	1	0.14514	0.8
